# SPOC1 modulates DNA repair by regulating key determinants of chromatin compaction and DNA damage response

**DOI:** 10.1093/nar/gks868

**Published:** 2012-10-02

**Authors:** Andreas Mund, Tobias Schubert, Hannah Staege, Sarah Kinkley, Kerstin Reumann, Malte Kriegs, Lauriane Fritsch, Valentine Battisti, Slimane Ait-Si-Ali, Anne-Sophie Hoffbeck, Evi Soutoglou, Hans Will

**Affiliations:** ^1^Heinrich-Pette-Institute, Leibniz-Institute for Experimental Virology, Department of General Virology, Martinistrasse 52, 20251 Hamburg, ^2^University Medical Center Hamburg, Clinic and Policlinic of Radiation Biology and Experimental Radiooncology, Laboratory of Radiobiology & Experimental Radiooncology, Martinistrasse 52, 20246 Hamburg, Germany, ^3^University Paris Diderot, Sorbonne Paris Cité, Laboratoire Epigénétique et Destin Cellulaire, UMR7216, Centre National de la Recherche Scientifique CNRS, 35 rue Hélène Brion, F-75013 Paris and ^4^Department of Cancer Biology, Institut de Génétique et de Biologie Moléculaire et Cellulaire (IGBMC), CNRS UMR 7104, INSERM U 596, Centre Universitaire de Strasbourg, 67404 Illkirch CEDEX, France

## Abstract

Survival time-associated plant homeodomain (PHD) finger protein in Ovarian Cancer 1 (SPOC1, also known as PHF13) is known to modulate chromatin structure and is essential for testicular stem-cell differentiation. Here we show that SPOC1 is recruited to DNA double-strand breaks (DSBs) in an ATM-dependent manner. Moreover, SPOC1 localizes at endogenous repair foci, including OPT domains and accumulates at large DSB repair foci characteristic for delayed repair at heterochromatic sites. SPOC1 depletion enhances the kinetics of ionizing radiation-induced foci (IRIF) formation after γ-irradiation (γ-IR), non-homologous end-joining (NHEJ) repair activity, and cellular radioresistance, but impairs homologous recombination (HR) repair. Conversely, SPOC1 overexpression delays IRIF formation and γH2AX expansion, reduces NHEJ repair activity and enhances cellular radiosensitivity. SPOC1 mediates dose-dependent changes in chromatin association of DNA compaction factors KAP-1, HP1-α and H3K9 methyltransferases (KMT) GLP, G9A and SETDB1. In addition, SPOC1 interacts with KAP-1 and H3K9 KMTs, inhibits KAP-1 phosphorylation and enhances H3K9 trimethylation. These findings provide the first evidence for a function of SPOC1 in DNA damage response (DDR) and repair. SPOC1 acts as a modulator of repair kinetics and choice of pathways. This involves its dose-dependent effects on DNA damage sensors, repair mediators and key regulators of chromatin structure.

## INTRODUCTION

Elevated SPOC1 RNA levels of the human *SPOC1* gene are associated with unresectable carcinomas and shorter survival in ovarian cancer patients, implicating a possible role in oncogenesis (1). The SPOC1 protein was recently also demonstrated to function in chromatin condensation and decondensation (2). The ability of SPOC1 to associate with, and structurally alter chromatin depends on its plant homeodomain (PHD) (2), predicted to bind to H3K4me2/3 (3). In a mouse *SPOC1* knockout model, SPOC1 protein expression was also recently shown to be indispensable for testis stem-cell differentiation and sustained spermatogenesis (4). These findings imply that SPOC1 plays a role in stem-cell maintenance, chromatin structure, and presumably also in oncogenesis. Considering these data and published evidence that chromatin structure plays a crucial role in radiosensitivity, DNA repair and mutation rates in cancer cells (5), we wanted to examine whether SPOC1 also has an impact on DNA damage response (DDR) and DNA repair.

Upon DNA damage, cells undergo a coordinated cascade of events which can result in DNA repair, which preserves genome stability and is critical in preventing tumorigenesis (6). DNA damage activates the DDR, which in turn induces cell-cycle arrest, and ensuing DNA repair or apoptosis. DDR proteins are hierarchically recruited to DNA damage sites and can be visualized as repair foci. In response to double-strand breaks (DSBs), the histone variant H2AX is phosphorylated (γH2AX) by the ATM kinase, which then associates with the MRN complex, triggering further chromatin alterations and the focal recruitment of additional DDR mediators, including 53BP1 (7). γH2AX and 53BP1 play distinct roles in DDR initiation and DNA repair of heterochromatin (8,9).

Two main DDR pathways drive repair of DSBs: homologous recombination (HR) and non-homologous end-joining (NHEJ) (10–12). HR requires a sister chromatid and can precisely repair DSBs primarily in the S phase of the cell cycle (13). In contrast, NHEJ functions throughout the cell cycle and is the main repair pathway for cells in G1 and G2. Since NHEJ does not require a homologous template and is error prone, it is potentially mutagenic (14).

Repair of DNA damage in euchromatin and heterochromatin are divergent due to different accessibility and requirements for DDR proteins. Approximately 10–25% of nuclear DNA is heterochromatic and characterized by H3K9me2/3 epigenetic marks (15,16). Recruitment of corepressors such as KAP-1 and the H3K9 KMTs (SUV39H1, SETDB1, G9A and GLP) to chromatin promotes its compaction by increasing histone H3K9 di and trimethylation, thereby potentiating the binding of chromodomain (CD) containing condensing ‘mediators’ such as HP1 (17,18).

In the event of DNA damage, the DNA repair machinery must overcome the physical barrier of heterochromatin (19–22). To achieve this, histones and chromatin-affiliated proteins are specifically post-translationally modified; then ATP-dependent chromatin remodeling factors are engaged to unwind the chromatin locally and facilitate access to the damaged DNA (6,23). For instance, regulation of H3K9me3 levels by several KMTs is intimately linked to the activation of ATM via TIP60-mediated acetylation at DSBs, a key process in coordinating DNA repair pathways (24). ATM helps locally to overcome the barrier to DDR signaling posed by heterochromatin by enhancing γH2AX expansion at repair foci, as shown with cells deficient in several heterochromatin components (25). This study also showed that heterochromatin has a substantial impact on the magnitude of ATM signaling and contributes to an inefficient G2/M checkpoint response.

Modulation of chromatin structure is a fundamental feature of DDR and DNA repair pathways (21,22). The heterochromatin building factors, KAP-1 and HP1, which are linked to H3K9 methylation, are important for, and affected by the DDR (26–28). After γ-IR, KAP-1 is phosphorylated at Ser824 by ATM, promoting chromatin decondensation at repair sites and enhancing cellular survival (29). It was previously demonstrated that phosphorylated KAP-1 (pKAP-1) is essential for repairing heterochromatin DSBs (9,30). Likewise, HP1 proteins are also phosphorylated in response to DNA damage, and appear important for recruiting DDR factors and dynamically reorganizing chromatin (27,31). Recently, HP1-α was shown to regulate KAP-1’s phosphorylation and accumulation at repair foci (32), linking both proteins to chromatin structural changes during DNA repair in heterochromatin.

Here we show that SPOC1 modulates DDR, NHEJ and HR repair pathways, and influences the radiosensitivity of cells by alternating key determinants of chromatin structure and interacting with DDR modifiers. Thus our data identify SPOC1 as a novel player in DDR and argue for its involvement in cancer development.

## MATERIALS AND METHODS

### Cell lines

U2OS, U2OS DR-GFP, U2OS19 ptight13 GFP-LacR, HEK293, CV1 cells, HeLa-S3 and C2C12 myoblasts were cultured according to the supplier’s instructions or as described previously (33–35).

SPOC1 inducible CV1 cell lines were generated with the Tet-On system (Invitrogen) by cotransfecting pcDNA6/TR Tet-repressor and pcDNA-4TO-FLAG-SPOC1-expression plasmids. Cells were selected with 300 µg/ml zeocin and 1.8 µg/ml blasticidine (Invivogen) before isolating single colonies. FLAG-SPOC1 expression was induced with 1 µg/ml doxycycline (Dox, Sigma). All cells were γ-irradiated with 2 Gy, unless indicated otherwise, using a ^137^Cs source.

Polyclonal HeLa-S3 cell lines stably expressing either FLAG-HA-tagged SETDB1 or pREV control vector (33) were analyzed before and after superinfection with lentiviruses (LeGO-iG2) expressing human SPOC1 wt or a PHD domain mutant (M246A + W255A). Detailed protocols for LeGO vector production and titration are reported elsewhere (36).

### Plasmids

Expression constructs for human SPOC1, SPOC1 mutants lacking amino acids 21–70 (deltaN), mutated in the PHD domain (M246A + W255A), or N-terminally FLAG-tagged SPOC1 constructs were generated in the pcDNA4-TO vector (Invitrogen). GST-fusion proteins for human SPOC1 full–length fragments, and deletion derivatives were produced with vector pGEX4T3 (GE Healthcare) in *Escherichia coli*. For the LUMIER assay, cDNAs for human SPOC1 and KAP-1 full-length and fragments were subcloned into pTREX-dest30nt-PrA (N-terminal protein A tag fusion) and/or pcDNA3-RL GW (N-terminal renilla luciferase fusion), provided by M. Koegel, DKFZ, Heidelberg.

### siRNA-mediated knockdown

CV1 cells were transfected with 100 nM SPOC1-specific siRNA (5′-UCACCUGUCCUGUGCGAAA-3′) or control siRNA (5′-AGGUAGUGUAAUCGCCUUG-3′) as described before (2) using Rotifect plus (Roth) and subsequently γ-irradiated.

### Drug treatments

Cells were treated with 100 ng/ml neocarzinostatin (NCS) (Sigma) for 15 min and afterwards cells were washed 2× with PBS and fixed at indicated time points. ATM inhibitor (KU55933) (37) (Tocris) was used at 20 μM.

### Antibodies

Indirect immunofluroescence and immunoblotting were performed using antibodies to γH2AX (05-636; Millipore), 53BP1 (NB100-304; Novus), β-actin (A-5441; Sigma), ATM (1549-1, Epitomics), pATM-S1981 (2152-1; Epitomics), Chk2 (sc-56297; Santa Cruz), pChk2-T68 (2661; Cell signaling), CtIP (D4) (sc-271339; Sigma), FLAG (F1804; Sigma), G9A (D141-3; Clinisciences), G9A (A300-933A; Bethyl), GAPDH (sc-32233; Santa Cruz), GLP (PP-B0422-00; R&D Systems), GLP (Bethyl; A301-642A), H3 (1326-1; Epitomics), H3K9me3 (07-442; Upstate), HP1-α (05-689; Upstate), HP1-β (ab10811; Abcam), HP1-γ (ab10817; Abcam), Tif1-β/KAP-1 (MAB3662; Millipore), pKAP-1-S824 (A300-767A-1; Bethyl), SETDB1 (ab12317; Abcam), SETDB1 (A300-121A; Bethyl), SUV39H1 (05-615; Upstate), SUV39H1 (07-550; Upstate). SPOC1 was detected with 6F6 rat monoclonal antibody (2) unless stated otherwise. Rabbit polyclonal anti-SPOC1 (CR56) was raised against amino acids 286–300 of human SPOC1 and rat monoclonal anti-SPOC1 (1D3) was raised against human SPOC1 fused to GST.

### Proliferation assay

CV1 cells and three different FLAG-SPOC1 (#14, #17, #23) expressing cell lines were seeded at a concentration of 2 × 10^5^ cells per 6-cm dish at Day 0. FLAG-SPOC1 cells were induced with Dox (1 µg/ml) or treated with ethanol on Day 0, which was replenished every 3 days. The cells were counted every 2–3 days in triplicates and cultivated over a period of 9 days.

### Extraction protocol, immunoblotting and immunoprecipitation

SPOC1 protein was extracted from cell pellets using either (i) a sodium dodecyl sulfate (SDS) total-cell lysate extraction buffer (50 mM Tris, pH 6.8, 2% SDS and 10% glycerol) or (ii) a fractionated lysate protocol. (i) In brief, the cell pellet (1 × 10^7^ cells) was resuspended in 100 μl of SDS total-cell lysis buffer supplemented with 1× protease inhibitor cocktail (EDTA-free complete, Roche), 1 mM NaF (Sigma), 1 mM NaVO_3_ (Sigma) and 1 mM PMSF (Calbiochem), heated at 99°C for 5 min, supplemented with Laemmli buffer and then reheated for an additional 5 min at 99°C before loading on a SDS–PAGE. (ii) The fractionated lysate protocol was performed as follows: soluble cellular proteins from ∼1 × 10^7^ cells were extracted with 100 μl of E1A buffer [150 mM NaCl, 50 mM HEPES, 0.1% NP-40 supplemented with 5 mM NaF, 5 µg/ml Trasylol® (Aprotinin), 250 µg/ml Pefablock® SC, 1 mM NaVO_3_, 1× PhosSTOP (Roche) and 1× complete (EDTA-free, Roche)] for 25 min on ice and were then centrifuged (15 min, 16 100*g*, 4°C). Chromatin-associated proteins were extracted from the remaining pellet in 100 μl of a chromatin extraction buffer [0.4 M NaCl, 50 mM HEPES, 1 % Triton® X-100, 0.1% NP-40, 2.5 mM MgCl_2_, 25 U of Benzonase (Novagen)] supplemented with 5 mM NaF, 5 µg/ml Trasylol® (Aprotinin), 250 µg/ml Pefablock® SC, 1 mM NaVO_3_, 1× PhosSTOP (Roche) and 1× complete (EDTA-free, Roche) for 25 min on ice and were then centrifuged (15 min, 16 100*g*, 4°C). SDS–polyacrylamide gel electrophoresis (PAGE) and immunoblotting were performed using standard protocols. Immunoblot signals were quantified by densitometry using ImageQuant LAS4000 and the ImageQuant TL Software or by using the ImageJ Software. Nuclear extracts used for immunoprecipitation of H3K9 KMTase complexes shown in Supplementary Figure S6 were prepared as described above, except that before use, we added Turbo DNase buffer and preheated for 1 min at 37°C before treating with 1.7 KU/µl Turbo DNase I (Ambion) for 20 min at 37°C (repeated twice). Finally we incubated the nuclear extract with 0.06 µg/µl RNase (Sigma) for 15 min on ice. Alternatively, the extract were preheated the samples for 1 min at 37°C in the presence of 1 mM CaCl_2_ and then treated with 0.0125 U/µl MNase at 37°C for 15 min. With 4 mM EDTA the MNase digestion was stopped and the extract incubated with or without 1% ethidium bromide (Etbr) on ice before use. Nuclear extracts were subjected to either G9A complex purification or used for immunoprecipitation of the endogenous SPOC1 protein, essentially as described (33).

### Immunofluorescence and ionizing radiation-induced foci quantification

Cell lines were fixed and immunostained as described before (2). In γ-irradiated cells the number of ionizing radiation-induced foci (IRIF) per cell was determined by automated high-content imaging (700–1000 cells/timepoint, BD Pathway 855 Bioimager) using the BD AttoVision-Software v1.6.

### DNA DSB repair reporter assay

H1299.EJ, H1299.GC and U2OS DR-GFP reporter cells were used to measure NHEJ and HR repair as described (35,38–41). For SPOC1 depletion, cells were cotransfected with 100 nM control or SPOC1-specific siRNA and 2 μg pDsRed-I-SceI-GR. For SPOC1 overexpression, 1 μg of pcDNA4/TO-SPOC1, SPOC1 M246A/W255A or SPOC1ΔNTD expression plasmid was cotransfected with 1 μg pDsRed-I-SceI-GR. 24 h after transfection, I-SceI site-specific DSBs were induced by addition of 100 ng/ml triamcinolone acetonide (TA, Sigma) and cells were harvested 24 h (NHEJ) or 48 h (HR) thereafter. The number of DNA repair indicating GFP positive cells were determined by FACS (FACScan, BD Biosiences) and normalized to the fraction of pDsRed-I-SceI-GR positive cells. For additional controls performed see Supplementary Information.

### Comet assay

The Comet assay was performed as described previously (42). SPOC1 knockdown cells and two different FLAG-SPOC1 (#17, #23) expressing cell lines were irradiated with 2 Gy on ice and harvested immediately. The olive tail moment was measured with the Comet analysis software VisComet 4.0 (Impuls Bildanalyse GmbH). Sixty comets were scored for each treated sample.

### LUMIER assay

The LUMIER assay was performed as described (43). In brief, the indicated KAP-1 and SPOC1 sequences fused to either protein A or luciferase were expressed in HEK293 cells. Protein A fusion proteins were purified from cell lysates, mixed with sheep anti-rabbit IgG-coated magnetic beads (Dynabeads M280, Invitrogen). Luciferase activity of copurified luciferase-tagged proteins was measured in four lysates and the corresponding washed beads. Lysates from cells expressing the luciferase fusion protein and a protein A dimer served as negative controls (nc). Normalized interaction signals were calculated Log(bound)/log(input) − log(bound nc)/log(input nc) and z-transformed by subtracting the mean and dividing by the standard deviation. The mean and standard deviation were calculated from large data sets of protein pairs not expected to interact. Normalized signal to noise ratios were calculated as (bound/input)/(bound nc/input nc) (44).

### GST pull-down

U2OS cells were lysed for 10 min with buffer 1 [150 mM NaCl, 25 mM HEPES, 2.5 mM MgCl_2_, 10% glycerol, 0.1% NP-40, 5 mM NaF, 250 µg/ml Pefablock® SC, 1 mM NaVO_3_ and 1× protease inhibitor cocktail (Roche)] and centrifuged at 16 000*g* at 4°C for 10 min. The supernatant was discarded and pellets were lysed for 30 min in ice-cold buffer 2 (150 mM NaCl, 25 mM HEPES, 2.5 mM MgCl_2_, 10% glycerol, 0.1% NP-40, 250 µg/ml Pefablock® SC and 1× Complete) + 25–50 units benzonase (Novagen) and cleared by centrifugation at 16 000*g* at 4°C for 10 min. Cell lysates were incubated with recombinant GST-fusion proteins produced in *E. coli* and bound to glutathione-S-transferase beads for 1–2 h at 4°C under rotation. Beads were washed three times with PBS and bound proteins were analyzed by SDS–PAGE and immunoblotting.

### Histone methylation assay

Histone methylation assay was monitored as previously described (33). Briefly, immunoprecipitated material was incubated with 5 μg of core histones (ref. 13-107, Upstate) and 1.5 μCi of Adenosyl-l-Methionine, S-[methyl-3H] (ref. NET155050UC, PerkinElmer) in a buffer containing 50 mM Tris pH 8.0, 100 mM NaCl, 1% NP40, 1 mM DTT and protease inhibitors (reaction volume 30 μl). The mixture was incubated 1 h at 30°C followed by SDS–PAGE analysis. The gel was stained by SimplyBlue kit (Invitrogen), and analyzed by autoradiography. IP efficiency was monitored by immunoblot (IB).

### Clonogenic survival assays

CV1 cells transfected with control or SPOC1-specific siRNA for 24 h and uninduced CV1 FLAG-SPOC1 cell lines were γ-irradiated with 0, 2, 4, 6 and 8 Gy. Cells were plated at different concentrations (125–1000 cells/6-cm plate) in triplicate and grown for 9–12 days. Colonies were fixed with methanol, stained with Giemsa (Merck) and counted.

### Statistical tests

Data significance was determined using an unpaired two tailed Student’s *t*-test.

## RESULTS

### SPOC1 is recruited to DSBs

**Figure 1.**
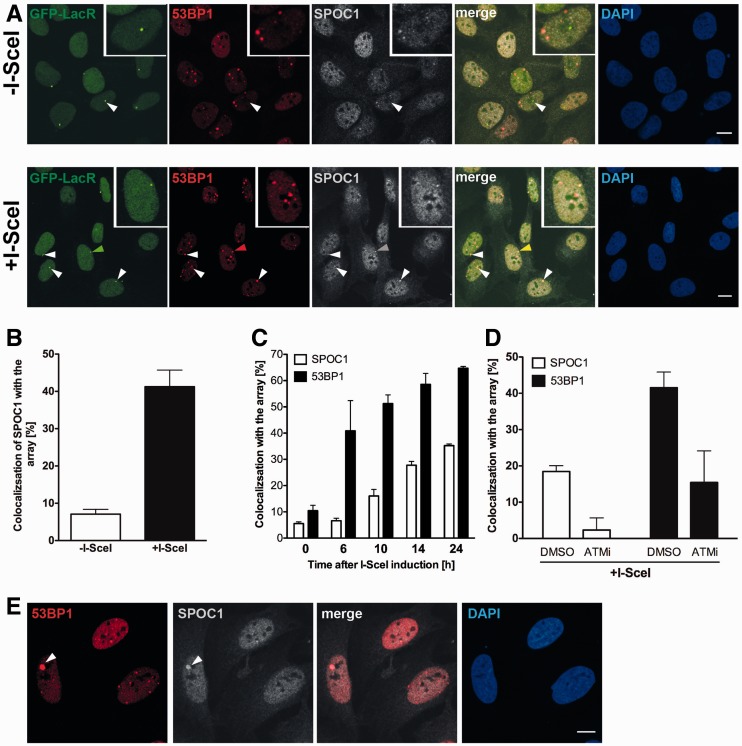
SPOC1 is recruited to I-SceI-induced DSBs where it colocalizes with 53BP1. (**A**) *In situ* immunofluorescence studies with U2OS19 ptight13 GFP-LacR cells with a stably integrated I-SceI cleavage site flanked by lac operator repeats reveals localization of the GFP-lac repressor protein (GFP-LacR) at the lac-operator DNA sequences in the nucleus before (−I-SceI) and 16 h after I-SceI-induced (+ I-SceI) DSB. In contrast, SPOC1 (white) and 53BP1 (red) are distributed throughout the nucleus in the absence of I-Scel, and partially colocalized in naturally occurring repair foci. After I-SceI cleavage to generate DSBs, SPOC1 and 53BP1 colocalize at distinct foci, including the cleaved DNA adjacent to DNA-bound GFP-LacR. Proteins were visualized by immunostaining and confocal microscopy. Scale bars = 10 μm. (**B**) Quantitative analysis of SPOC1 recruitment to the 53BP1 and GFP-LacR positive lacO array before and 16 h after I-SceI induction. (**C**) Monitoring of the kinetics of SPOC1 and 53BP1 recruitment to DSBs between 0 and 24 h post-I-SceI induction. (**D**) Quantifying ATM kinase inhibitor-mediated effects on recruitment of SPOC1 and 53BP1 to DSBs as evident 16 h after I-SceI-induced cleavage. (**E**) SPOC1 and 53BP1 colocalize at a large discrete endogenous repair focus as observed in some non-irradiated U2OS cells by immunostaining. Scale bar = 10µm.

To determine whether SPOC1 is specifically recruited to DSBs, we used the reporter cell line U2OS19 ptight13 GFPlacR, which carries a stably integrated lac-operator array upstream of a I-SceI restriction site followed downstream by an array of Dox response elements (34). In addition, this cell line contains two transgenes, one constitutively expressing GFP-LacR, and the other expressing Dox-inducible HA-I-SceI endonuclease in most cells, but due to leaky expression also Dox-independently in a few cells. The I-SceI site is converted into DSBs in most cells after Dox-induced I-SceI endonuclease expression. The GFP-LacR protein binds to lac-operator sequences, forming a single green dot independently of I-SceI restriction site cleavage by the endonuclease (45) (Figure 1A). In these non-treated cells endogenous SPOC1 protein was concentrated at many, but not all endogenous 53BP1-positive repair foci (Figure 1A). Prior to Dox-induced I-SceI expression, SPOC1 did not accumulate at GFP-LacR stained LacO arrays devoid of focal 53BP1 staining (Figure 1A, -I-SceI-). In contrast, 16 h after Dox-induced I-Sce-I expression SPOC1 localized to a large number of foci double-labeled by GFP-LacR/53BP1 (Figure 1A, +I-Sce-I). Determining the frequency of SPOC1 foci accumulation at GFP-LacR/53BP1 double-labeled repair foci before and 16 h after Dox-induced I-SceI-expression revealed an increase from 8% to 40% (Figure 1B). Detailed analysis of the recruitment kinetics showed rather late accumulation of SPOC1 at these foci compared to 53BP1.

We also examined whether ATM kinase activity is required for recruiting SPOC1 to I-SceI-induced DSBs. Inhibition of ATM kinase almost completely reduced SPOC1 recruitment to repair foci 16 h after Dox-induced I-SceI expression (Figure 1D). Next, we examined whether SPOC1 is present at large (2–3 μm) 53BP1 containing domains, also called OPT domains, which occur occasionally in G1 cells (46,47). OPT domains are frequently located at the periphery of nucleoli (48) and carry features of DSBs in condensed chromatin regions (46,47). In fact, in non-treated U2OS cells we observed a small number of discrete 53BP1/SPOC1 positive foci that fulfill the criteria of OPT domains (Figure 1E). Taken together, these data demonstrate that SPOC1 is recruited to I-SceI-induced DSBs in an ATM-dependent manner, as well as to OPT domains that arise at sites of DNA damage.

**Figure 2.**
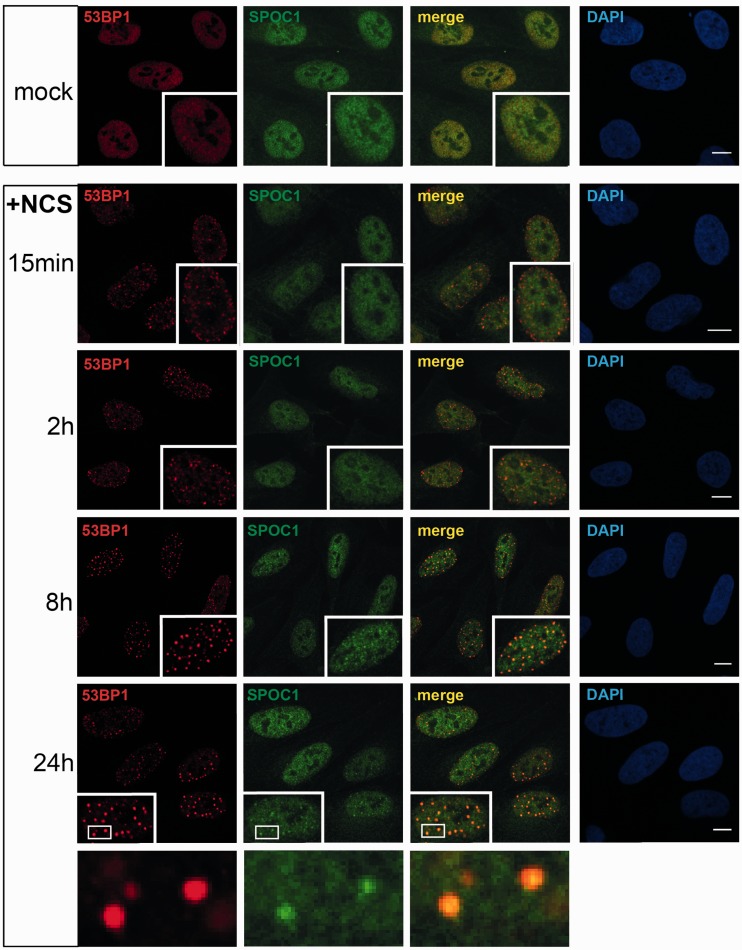
Comparative kinetics of 53BP1 and SPOC1 recruitment to radiomimetic drug NCS-induced DSBs. U2OS cells fixed before (mock) or at the indicated times post-treatment (+NCS), immunostained for SPOC1 (green) and 53BP1 (red) and analyzed by confocal microcopy. At the earliest observation period after NCS treatment (15 min) a large number of small 53BP1 stained DSB repair foci emerged, but were not detectably stained by SPOC1. Between 2 to 24 h post-NCS treatment the number of small 53BP1 stained foci gradually, then almost completely disappeared, concomitant with emerging larger repair foci. At around 8 h post-NCS treatment most of the large 53BP1-stained repair foci were also strongly stained by SPOC1, suggesting SPOC1 is recruited to and accumulates at DSBs in heterochromatin known for their slow repair kinetics. Scale bars = 10µm.

Furthermore, using the radiomimetic drug NCS we could show that SPOC1 is recruited to DSBs induced by the drug, although its accumulation at repair foci is rather late compared to 53BP1 (Figure 2). These data and the reported similar repair kinetics of γ-IR- and NCS-induced DSBs (30) provide evidence for SPOC1 accumulation at naturally occurring replication stress-, I-SceI-, radiomimetic drug- and γ-IR-induced repair foci with slow repair kinetics, a well documented feature of DSB repair in highly compacted chromatin regions (28). The findings and the known function of SPOC1 in chromatin compaction/decondensation are consistent with the assumption that SPOC1 contributes to structural changes in chromatin required for repair of DSBs in heterochromation.

### SPOC1-expression levels influence the DDR after γ-irradiation

**Figure 3.**
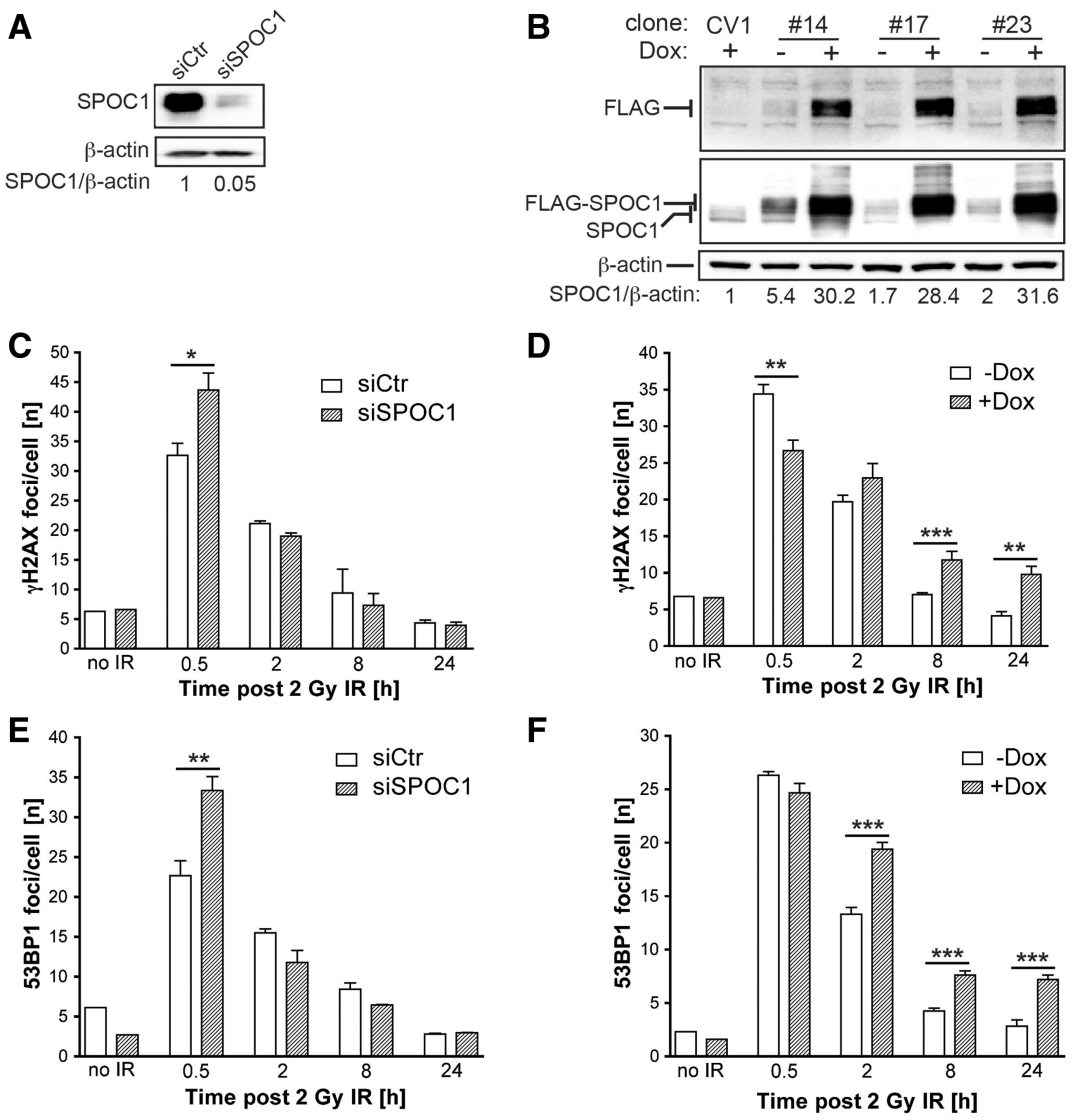
SPOC1 dose-dependent modulation of DDR after γ-IR. SPOC1 expression in CV1 cell lines was determined by immunoblotting cell extracts with the indicated antibodies (Supplementary Information). (**A**) SPOC1 protein levels 72 h post-siRNA transfection; (**B**) Endogenous and FLAG-SPOC1 expression in three stably transformed CV1 cell lines (clones #14, #17 and #23) pre- or 24 h post-Dox treatment. Signals were quantified by densitometry. (**C–F**) IRIF visualized by indirect immunofluorescence of CV1 cells (unpublished observations) were counted by automated imaging at various times after γ-irradiation. (C, E) γH2AX and 53BP1 foci in CV1 cells 72 h after siRNA-mediated reduction of SPOC1 (siSPOC1) compared to control cells (siCtr). (D, F) γH2AX and 53BP1 IRIF in CV1 clone #14 before and after Dox-induced (24 h) expression of FLAG-SPOC1. The IRIF of 700–1000 cells were counted in each of three independent radiation experiments and at different time points, and are represented as the mean/cell ± SEM. *P ≤ 0.05, **P ≤ 0.01, ***P≤ 0.001.

We next investigated the role of SPOC1 in DDR and repair, and measured the effect of SPOC1 expression on the formation of γH2AX and 53BP1 foci post-γ-IR with 2 Gy. Expression of endogenous or exogenous FLAG-tagged SPOC1 in CV1 cell lines was either diminished by specific siRNA (Figure 3A) or induced by Dox (Figure 3B). Thirty minutes post-γ-IR, the number of γH2AX and 53BP1 foci per cell counted in SPOC1-deficient cells was significantly more than in control cells (Figure 3C and E). At later time points during DNA repair, we detected little difference between numbers of γH2AX and 53BP1 foci, whereas the repair kinetics were faster (1.5-fold γH2AX and 1.9-fold 53BP1) between 30 min and 2 h, but not between 8 and 24 h later (Figure 3C and E).

In contrast, SPOC1 overexpression significantly reduced the number of γH2AX, but not 53BP1 foci (Figure 3B, D and F) 30 min post-γ-IR. Interestingly, the γH2AX and 53BP1 foci in SPOC1 overexpressing cells decreased, in other words were repaired at a significantly slower rate (3.1-fold γH2AX and 2.7-fold 53BP1) between 30 min and 24 h post-γ-IR compared to control cells (Figure 3D and F). These findings suggest that increased SPOC1 expression interferes with the kinetics of IRIF formation/disappearance and DNA repair.

To investigate whether the SPOC1 levels affect the extent of inflicted DNA damage we performed Comet assays using cells immediately after γ-irradiation with 2 Gy kept on ice. Neither SPOC1 knockdown nor SPOC1 overexpression revealed significant differences in the initial number of DSB (Supplementary Figure S1A and C). In the context of this experiment we also analyzed by FACS whether altered SPOC1 levels are associated with detectable changes in the cell cycle. We did not see obvious differences between control cells, SPOC1-depleted and SPOC1 overexpressing cells (Supplementary Figure S1B and D). Taken together, up and downregulation of SPOC1 expression does not alter the radiosensitivity of chromatin but inversely modulates the kinetics of IRIF formation and DNA repair.

### SPOC1 inversely affects NHEJ and HR activity

**Figure 4.**
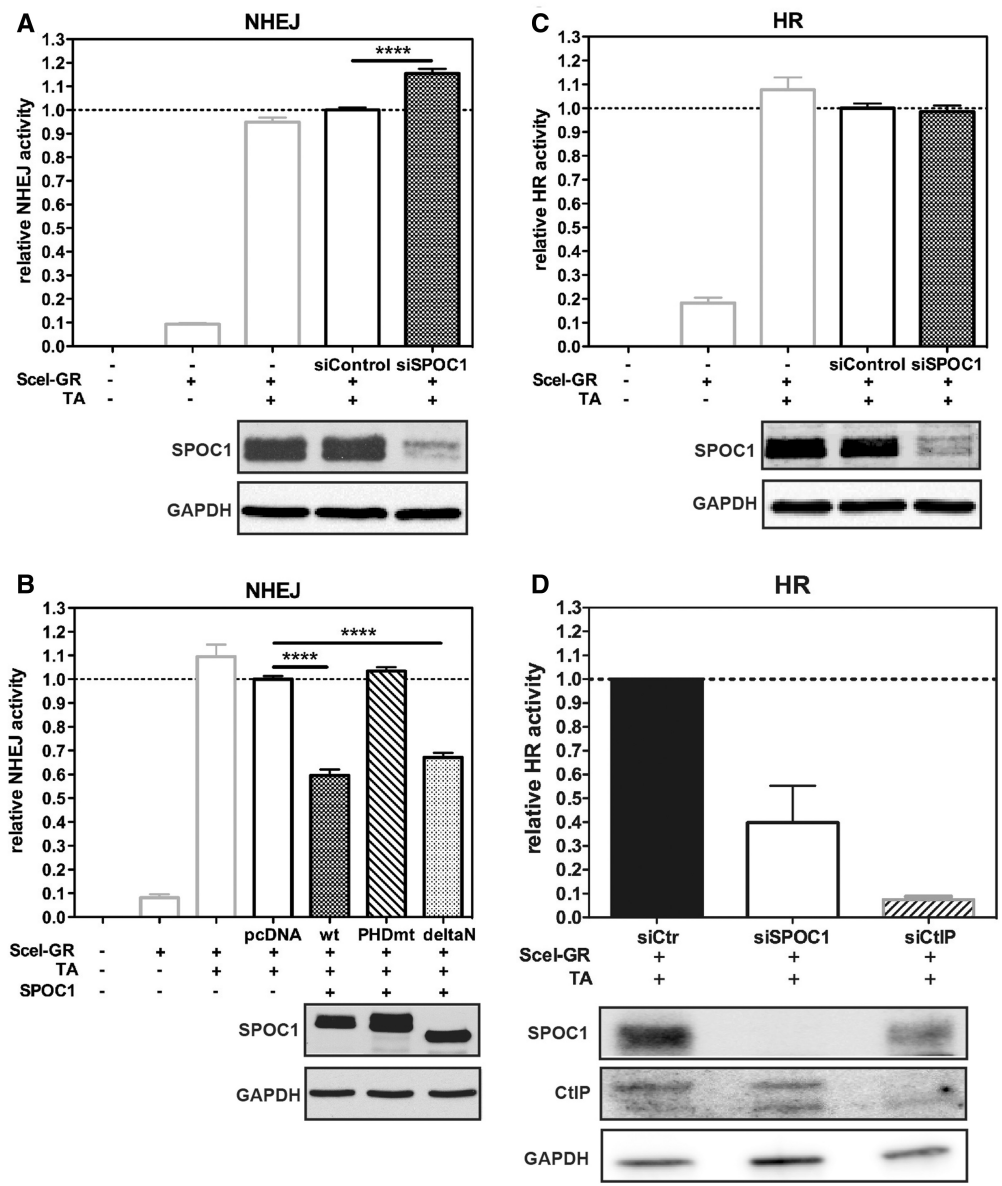
SPOC1-expression levels modulate NHEJ activity. (**A**) siRNA-mediated SPOC1 depletion increases NHEJ activity in H1299.EJ reporter cells compared to control siRNA-transfected cells as quantified by flow cytometry detecting EGFP expression. NHEJ activity is only evident after I-Scel-mediated cleavage in cells transfected with a plasmid expressing cytoplasmic SceI fused to glucocorticoid receptor ligand-binding domain (SceI-GR), plus TA to translocate the fusion protein into the nucleus. (**B**) Enhanced expression of SPOC1wt and a SPOC1 N-terminal domain deletion (deltaN), but not a PHD-deficient derivative (PHDmt) strongly reduced NHEJ activity when analyzed and quantified as in (A). (**C**) DNA repair activity by HR in H1299.GC reporter cells after SPOC1 siRNA or control siRNA transfection, quantified as in (A), were similar. (**D**) HR-mediated DNA repair activity in U2OS-DR-GFP reporter cells was reduced by >60% after siRNA-induced SPOC1 knockdown compared to control siRNA treated cells. Knockdown of CtIP, a protein critical for the initial steps of HR, reduced HR activity by ∼90% compared to the control siRNA transfected cells. All data are from triplicates derived from three independent experiments (±SEM) ****P ≤ 0.0001. (A–D) Successful SPOC1 and CtIP knockdown as well as equivalent expression levels of SPOC1 wt and mutant proteins were confirmed by immunoblotting of cell extracts using anti-SPOC1, anti-CtIP and anti-GAPDH antibodies.

To evaluate which DNA DSB repair pathways SPOC1 influences we used the well-established H1299.EJ (NHEJ) and H1299.GC (HR) reporter cell lines (39,41). Both cell lines contain chromosomally integrated EGFP that is only expressed after I-Scel-mediated cleavage and repair (Supplementary Figure S2). By quantifying *in vivo* EGFP expression, we observed ∼20% more NHEJ activity after SPOC1 knockdown, but no change in HR activity (Figure 4A and C). In contrast, SPOC1 overexpression in H1299.EJ cells decreased NHEJ activity by 40% compared to control cells, as did a SPOC1 N-terminal domain deletion (deltaN) (Figure 4B). Interestingly, this inhibitory effect was not observed with SPOC1 containing two critical amino acid mutations in the H3K4me3-binding pocket (PHDmt). The effect of SPOC1 on NHEJ is specific since EGFP-reporter expression was not significantly changed by transfection of H1299.EJ cells with SPOC1-expression vectors or siRNA (Supplementary Figure S3). These data indicate that NHEJ DNA repair activity is modulated by SPOC1 in a dose- and PHD-dependent manner

In contrast, SPOC1 overexpression strongly suppressed EGFP reporter expression in H1299.GC cells (unpublished observations), making it impossible to evaluate specific effects of SPOC1 overexpression on HR activity in this system. Since we supposed that a SPOC1-mediated effect on HR may be cell context dependent, we also studied this in the well-established and frequently used HR-reporter cell line U2OS DR-GFP (35). Compared to the H1299.GC cell line, this reporter cell line differs in p53 status and the I-SceI chromosomal integration sites and its associated chromatin structure. Using the U2OS DR-GFP cell line we observed ∼60% less HR-mediated gene conversion upon SPOC1-depletion. As a positive control we induced siRNA-mediated depletion of CtIP, a protein critical for the initial step of HR (49) (Figure 4D) and observed that HR activity decreased by ∼90%. These data and no apparent effect of SPOC1 depletion in the H1299.GC cell line (Figure 4C) indicate a cell context-dependent role of SPOC1 in HR. Combined with the increase of NHEJ induced by SPOC1 knockdown as observed in the NHEJ H1299.EJ reporter cell line, the findings suggest that SPOC1 can shift the balance between NHEJ and HR.

### SPOC1 influences radiosensitivity after γ-IR

**Figure 5.**
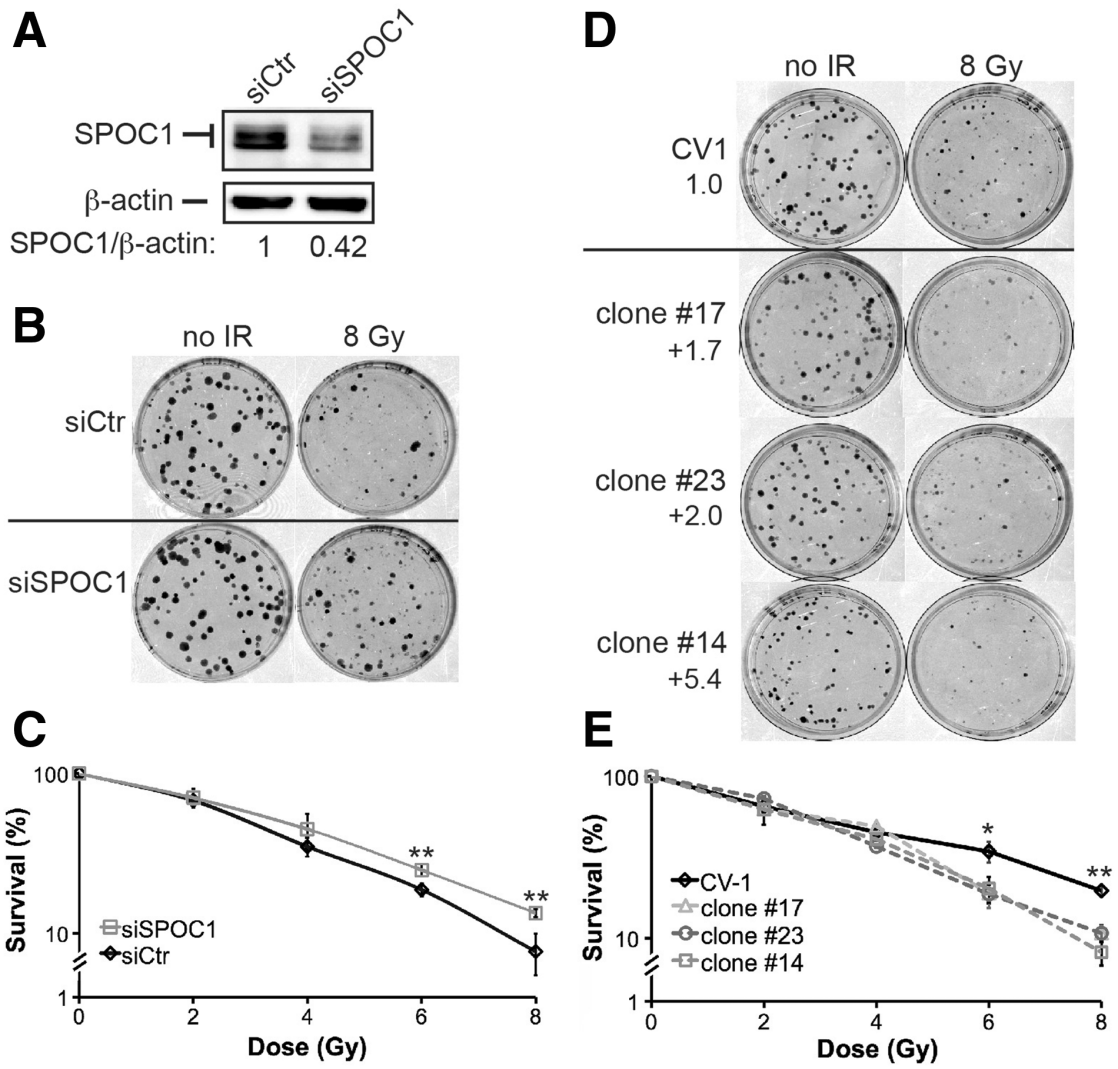
SPOC1 protein expression levels affect cell survival after γ-IR. (**A**) Immunoblot analysis shows that SPOC1 levels in SPOC1 siRNA transfected CV1 cells are reduced by 58% compared to control siRNA-transfected cells as analyzed by densitometry. These cells were used for clonogenic survival assays (see below). (**B**) Colony formation assays: SPOC1-siRNA treated cells formed more colonies than control siRNA transfected cells when analyzed after 8 Gy γ-IR and 9 days post-plating, indicating higher radioresistence. (**C**) Survival curves based on clonogenic growth of CV1 cells transfected with SPOC1-siRNA or control siRNA, analyzed after 0–8 Gy γ-IR and up to 9 days after plating. SPOC1 knockdown results in increased radioresistance. (**D**) Colony formation assays: different SPOC1 overexpressing (1.7- to 5.4-fold) cell lines formed less colonies than control cells when analyzed after 8 Gy γ-IR and 12 days post-plating, indicating higher radiosensitivity. (**E**) Survival curves based on clonogenic growth of SPOC1 overexpressing (1.7- to 5.4-fold) or control cells after 0–8 Gy γ-IR and analyzed 12 days after plating. SPOC1 overexpression results in lower radioresistance. Cells were plated in two different densities and in triplicates. Mean values ± SD are given. *P ≤ 0.05; **P ≤ 0.01.

To clarify the biological relevance of SPOC1-modulated DDR and DSB repair, we analyzed the radiosensitivity of CV1 cells lacking or overexpressing SPOC1 by clonogenic survival assays. CV1 cells with reduced SPOC1 levels formed more colonies than control cells after γ-IR (Figure 5A–C).

In contrast, CV1 cells stably overexpressing SPOC1 protein (clones in Figure 3B) formed fewer colonies after γ-IR (Figure 5D and E). Proliferation of non-irradiated cells depleted for SPOC1 did not significantly differ from control cells, and was marginally slower in cells with enhanced SPOC1 expression (Supplementary Figure S4). These data imply that SPOC1 modulates cellular radiosensitivity.

### SPOC1 modulates DDR and heterochromatin building factors

**Figure 6.**
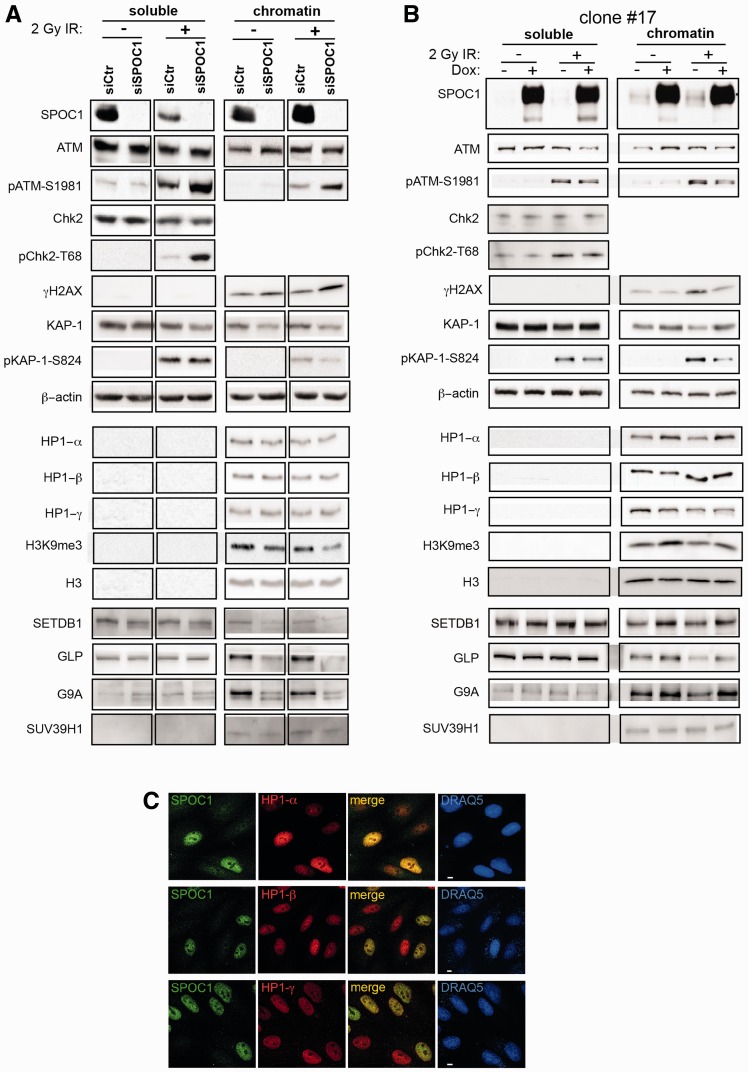
SPOC1 levels modify expression, modification and localization of proteins involved in chromatin (de)compaction and DDR. Immunoblots of soluble and chromatin-rich fractions from untreated (−) and irradiated cells collected 30 min post-2 Gy γ-IR (+) analyzed with Abs against the indicated proteins. (**A**) SPOC1-siRNA transfected cells show less SPOC1 protein than control CV1 cells. (**B**) Dox-treatment strongly enhances SPOC1 levels in CV1 cell line clone #17. (A and B) Immunoblot signal intensities for different cellular proteins detected in the corresponding cellular lysates show specific changes in expression of some but not all, depending on the soluble or chromatin fraction, SPOC1-expression levels and/or γ-IR. (**C**) Confocal immunofluorescence analysis of mixed CV1 cells over and underexpressing SPOC1 immunostained for HP1-α (top), HP1-β (middle) and HP1-γ (lower panels) only showed enhanced HP1-α expression in SPOC1-overexpressing cells. SPOC1 was immunostained with antibody CR56, the HP1 proteins with the antibodies given in ‘Materials and Methods’ section, and DNA was stained by DRAQ5. Scale bars = 5 μm.

SPOC1’s ability to alter chromatin compaction, DDR, DNA repair and radiosensitivity could be linked. To identify SPOC1 regulated factors with functions in more than one of these processes, we explored SPOC1- and γ-IR-dependent changes in expression, subnuclear localization and post-translational modifications of candidate nuclear proteins. Cell extracts from control or SPOC1-depleted CV1 cells either mock treated or γ-irradiated with 2 Gy and collected 30 min later were subfractionated into soluble and chromatin-bound proteins and analyzed by immunoblotting (Figure 6A). Upon γ-IR of the control cells SPOC1 protein levels decreased (2.1-fold) in the soluble fraction and increased (1.3-fold) in the chromatin-rich fraction (Figure 6A, siCtr). Furthermore, SPOC1 depletion appeared to increase phosphorylation-dependent activation of ATM (pATM-S1981 (soluble 1.7-fold and chromatin-associated 1.9-fold), Chk2 (pChk2-T68, 3.6-fold) and γ H2AX (1.6-fold) in irradiated cells, whereas total levels of ATM and Chk2 remained unchanged (Figure 6A). Increased pATM-S1891 and pChk2-T68, phosphorylated by activated ATM (50,51), after SPOC1-depletion indicates elevated DDR, and is consistent with more IRIF, enhanced DNA repair kinetics and increased NHEJ activity observed in the previous experiments (Figures 3C, E, G and 4A).

ATM-mediated KAP-1 phosphorylation at serine 824 leads to chromatin decondensation, an important process in DDR. Therefore, we evaluated whether SPOC1 affects KAP-1 expression and phosphorylation, as well as that of its binding partner HP1, which targets H3K9me3. We detected 30% less chromatin-bound KAP-1 protein in SPOC1-deficient cells compared to controls cells (Figure 6A). As expected, phosphorylated KAP-1 (pKAP-1-S824) was only detected after γ-IR, with levels related to those of total KAP-1 protein (Figure 6A). SPOC1 depletion did not alter expression of any isoforms of HP1, although levels of H3K9me3 were reduced (by 32% and 45% in non-irradiated and irradiated cells, respectively) (Figure 6A).

Several H3K9 methylation enzymes (SETDB1, GLP, G9A and SUV39H1) are present in large multisubunit complexes that promote H3K9 trimethylation (33). The amounts of three out of the four chromatin-associated enzymes decreased upon SPOC1 depletion both pre- and post-γ-IR (Figure 6A) explaining the decrease in H3K9me3. Less H3K9 KMTs, H3K9me3, and chromatin-bound KAP-1 proteins are likely linked to SPOC1 depletion-induced chromatin relaxation (2) and probably enhance accessibility of damaged sites for factors involved in DDR and repair.

Conversely, we tested whether SPOC1 overexpression affects the same proteins. SPOC1 overexpression slightly reduced both chromatin-bound ATM and activated ATM (pATM-S1981) after γ-IR (Figures 6B and Supplementary Figure S5). The ATM-dependent phosphorylation of KAP-1 at S824, as well as that of H2AX, was strongly reduced in SPOC1 overexpressing cells (by 60% and 30%, respectively), whereas total KAP-1 levels increased slightly (1.7-fold, Figures 6B and Supplementary Figure S5A). Reduced ATM activity is also in agreement with the lower number of γH2AX foci and the globally reduced γH2AX level observed after SPOC1 overexpression (Figures 3D and 6B). These data argue that SPOC1 overexpression impedes DDR and repair by promoting chromatin compaction.

To identify possible factors involved in SPOC1-induced chromatin compaction that may also affect DDR, we analyzed expression levels of the heterochromatin markers HP1 (all isoforms), H3K9me3 and KAP-1. Independent of γ-IR, levels of HP1α, H3K9me3, and also KAP-1 increased in the chromatin fraction 1.4-, 1.7- and 1.4-fold, respectively, after SPOC1 overexpression, whereas those of HP1-β and HP1-γ did not (Figure 6B, C and Supplementary Figure S6). Elevated levels of HP1-α, H3K9me3 and KAP-1 are consistent with the reported ability of SPOC1 to induce chromatin compaction (2).

As above, we then evaluated expression levels of the same H3K9 KMTs after SPOC1 overexpression. Now, chromatin-bound levels of SETDB1, GLP and G9A, but not SUV39H1, increased 1.2- to 1.4-fold in irradiated and non-irradiated SPOC1-overexpressing cells (Figure 6B and Supplementary Figure S5A). Thus, SPOC1 overexpression increases chromatin-associated H3K9 KMT and H3K9me3 levels, exactly the opposite of SPOC1 downregulation. Elevated H3K9me3 may promote chromatin compaction by recruiting appropriate epigenetic readers and their binding partners, including HP1-α and KAP-1, whereas lower H3K9me3 levels may have the reverse effect. In summary, the observed SPOC1-dependent changes in phosphorylation of ATM, Chk2 and KAP-1 could all contribute to SPOC1’s impact on DDR and DNA repair.

### SPOC1 interacts with KAP-1 and H3K9 KMTs

**Figure 7.**
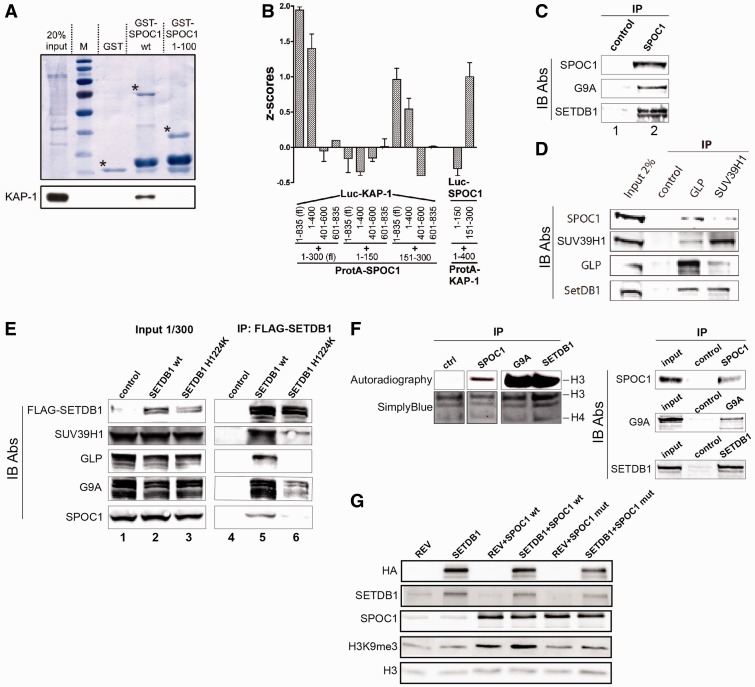
SPOC1 protein interacts with KAP-1 and enzymatically active H3K9 KMTs. (**A**) In GST-pulldown experiments with GST-SPOC1 wt, GST-SPOC1 (amino acids 1–100) or GST alone and chromatin extracts from U2OS cells, only the GST-SPOC1-full-length protein precipitated the KAP-1 protein, as detected on the immunoblot (lower panel). Amounts of GST proteins used were visualized by Amido black staining of the blots (upper panel). Asterisks mark the positions of full-length GST-proteins. (**B**) LUMIER-assay for mapping the KAP-1/SPOC1 interaction. The indicated combinations of SPOC1 and KAP-1, ProteinA (ProtA) and luciferase (Luc) fusion proteins were coexpressed. Luc activity coprecipitated with IgG-beads was measured (Z-scores). Significant KAP-1-interaction is only evident between both full-length proteins, or the C-terminal half of SPOC1 and the N-terminal 400 amino acids of KAP1, as detected by significant Luc activity in the corresponding pellets. *Z*-score ≥ 0.5 indicates an interaction. Mean values ± SD of three independent experiments each performed in triplicate are given. (**C**) Immunoprecipitation of SPOC1 from HeLa nuclear extracts with anti-SPOC1 (lane 2) or with control antibodies (lane 1) and analysis of pelleted material by immunoblotting shows specific coprecipitation of G9a and SETDB1 with SPOC1. (**D**) Immunoprecipitation (IP) of endogenous GLP or SUV39H1 from C2C12 cell extracts and immunoblotting with the indicated Abs (IB Abs) against GLP (PP-B0422-00), SUV39H1 (07-550), SETDB1 (ab12317) and SPOC1 (6F6). Unlike the control Ab, GLP and SUV39H1 Abs precipitated all associated KMTs as well as SPOC1. (**E**) Nuclear extracts of control C2C12 cells (lane 1) or cells expressing HA-FLAG-tagged SETDB1 wt (lane 2) or SETDB1 H1224K mutant (lane 3) were analyzed on immunoblots (IB Ab.) with antibodies against FLAG (F1804), G9A (D141-3), GLP (PP-B0422-00), SUV39H1 (07-550) and SPOC1 (6F6). After anti-FLAG-SETDB1 immunoprecipitation (IP) the immunoblot detected specific coprecipitation of SPOC1 by wt SETDB1 (lane 5) but much less by enzymatically inactive SETDB1 H1224K (lane 6). (F) *In vitro* analysis of histone H3 methlylation by proteins immunoprecipitated by control, SPOC1, G9A, or SETDB1 antibodies. Left panel: autoradiography and SimplyBlue staining of the assay products separated by SDS–PAGE. Right panel: immunoblot which demonstrates that SPOC1, G9A and SETDB1 were immunoprecipitated specifically from the HeLa nuclear extracts. (**G**) In total lysates from HeLa-S3 cell lines constitutively expressing HA-FLAG-SETDB1 (HA-SETDB1), lentiviral-mediated SPOC1wt expression elevates H3K9me3 levels compared to control cells (REV), as detected by immunblotting with Abs against the indicated proteins (left). The SPOC1 PHD mutant (SPOC1mut) unable to bind to H3K4me3 only marginally increased H3K9me3 levels compared to SPOC1 wt.

The observed correlation between chromatin-associated KAP-1 and SPOC1 levels (Figure 6A and B) may be due to KAP-1 interacting with SPOC1. To provide direct evidence for a SPOC1/KAP-1 interaction we performed GST pull-down experiments (Figure 7A). Full-length GST-SPOC1 protein precipitated KAP-1, unlike GST alone or the N-terminal 100 amino acids of SPOC1 (GST-SPOC1 1-100; Figure 7A).

To independently verify, and further map the SPOC1/KAP-1 interaction, we used a LUMIER-assay (Figure 7B). Full-length or C-terminal SPOC1 fused to protein A (ProtA-SPOC1; amino acids 1–300, 150–300) pulled down luciferase (Luc) fused to full-length or N-terminal KAP-1 (Luc-KAP-1; amino acids 1–835, 1–400), but not C-terminal derivatives (Luc-KAP-1; amino acids 401–600, 601–835), while protein A-tagged N-terminal KAP-1 (ProtA-KAP-1; amino acids 1–400) efficiently pulled down C-terminal SPOC1 (Luc-SPOC1; amino acids 150–300) (Figure 7B). These results indicate a specific interaction between the N-terminal 400 amino acids of KAP-1 and the C-terminal 150 amino acids of SPOC1. The interaction with SPOC1 may affect KAP-1 stability, phosphorylation, and/or its recruitment to chromatin before and after γ-IR.

We then investigated whether SPOC1 interacts with components of the H3K9 KMT multiprotein complex (33,52). Endogenous SPOC1 from HeLa nuclear extracts with anti-SPOC1 antibody but not with control antibody coprecipitated both G9A and SETDB1 as revealed by immunoblotting (Figure 7C). In addition, both endogenous SPOC1 and SETDB1 were specifically immunoprecipitated with antibodies against GLP or SUV39H1 (Figure 7D).

Previously described cell lines (52) expressing either FLAG-tagged SETDB1 wt, or an enzymatically inactive SETDB1 (SETDB1 H1224K), were used to further evaluate the SPOC1/H3K9 KMT interaction. With anti-FLAG antibodies wt SETDB1 efficiently coprecipitated endogenous SPOC1 and all three tested H3K9 KMTs (GLP, G9A and SUV39H1), compared to the less efficient coprecipitation by the mutant SETDB1 protein (Figure 7E). These data imply that SPOC1 interaction with H3K9 KMT molecular complexes partly depends on SETDB1 enzymatic activity. The nuclear extracts used for coimmunoprecipitation and protein complex immunoaffinity purification experiments were pretreated with DNase with or without added RNase or Etbr and analyzed for residual DNA by agarose gel electrophoresis (see ‘Materials and Methods’ section and Supplementary Figure S6). These experiments exclude that coIPs of SPOC1 and H3K9 KMTs are mediated by DNA.

An *in vitro* histone methlylation assay was then used to check if SPOC1-bound H3K9 KMTs are enzymatically active. The addition of chicken core histones and radiolabeled Adenosy-l-Methionine-[methyl-3H] to SPOC1 immunoprecipitated proteins resulted in methylation of histone H3, as also observed with immunoprecipitated G9A or SETDB1 (Figure 7F). This result shows SPOC1-bound H3K9 KMTs are active.

SPOC1 overexpression-induced increase of only chromatin-bound H3K9 KMT (Figure 6B) might depend on SPOC1’s binding to chromatin. Therefore, we analyzed H3K9me3 levels in total lysates from HeLa-S3 cell lines expressing either endogenous or ectopically enhanced levels of SETDB1, with or without lentivirally expressed SPOC1 wt or the SPOC1-PHD mutant. Neither expression of SETDB1 nor SPOC1-PHD mutant alone increased H3K9me3 levels (Figure 7G). In contrast, extra SPOC1 wt significantly enhanced H3K9me3 levels (Figure 7G), as was seen in CV1 cells (Figure 6B). H3K9me3 levels were even higher when both SPOC1 and SETDB1 were overexpressed (Figure 7G). However, these coexpression conditions did not detectably increase SETDB1 protein levels, suggesting instead that SPOC1 stimulates SETDB1 activity. In summary, these data demonstrate that SPOC1 overexpression can increase both chromatin-associated levels and activity of H3K9 KMTs, thus enhancing H3K9 trimethylation.

## DISCUSSION

Here, we provide the first evidence that SPOC1-expression levels have an impact on DDR, DNA repair kinetics and pathway choice, as well as on cellular radiosensitivity. We propose a molecular mechanism that is predominantly deduced from the following observations: (i) ATM kinase-dependent recruitment of SPOC1 to DSBs; (ii) SPOC1 accumulation at endogenous and experimentally induced repair foci with slow repair kinetics; (iii) SPOC1-dependent differences in the number of IRIFs formed early after γ-IR; (iv) altered kinetics of γ-IR-inducible DSB repair; (v) altered cellular radiosensitivity; (vi) the ability of SPOC1 to modulate dose-dependently both NHEJ and HR activity and (vii) SPOC1’s interaction with, and modulation of proteins that have intertwined functions in regulating chromatin structure as well as DDR and DNA repair.

**Figure 8.**
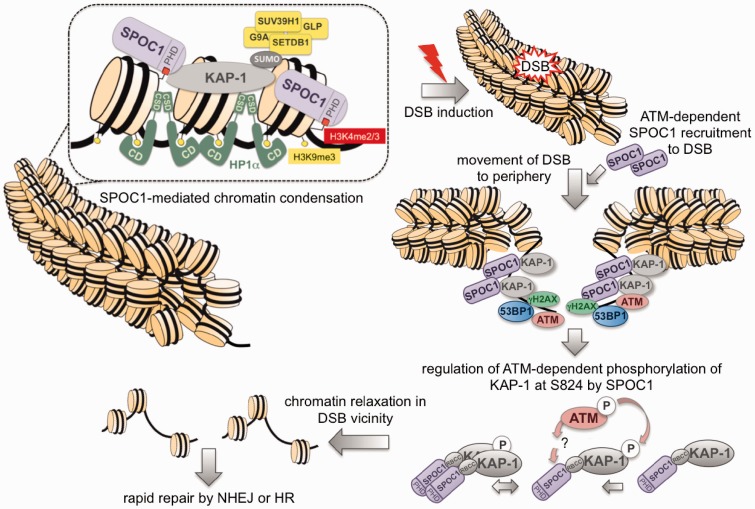
Model: mechanisms of SPOC1-associated chromatin compaction and modulation of DDR at DSBs. The interaction of SPOC1 with heterochromatin building factors KAP-1, HP1 and H3K9 KMTs and its binding to chromatin via H3K4me2/3 and/or other factors can induce chromatin compaction. DNA damage-induced DSBs in heterochromatin activate ATM kinase. This results in pan-nuclear KAP-1S824 phosphorylation and concomitant ATM recruitment to repair foci, and also to increasing accumulation at repair foci. SPOC1 is also ATM-dependently recruited to DSBs and accumulates detectably only at heterochromatic repair foci after γ-H2AX expansion and 53BP1 accumulation. The KAP-1S824 phosphorylation required for chromatin relaxation at DSBs in heterochromatin and for DNA repair is regulated by SPOC1. For more details see text.

SPOC1 binding to chromatin and its PHD-dependent ability to alter chromatin compaction (2), as well as its predicted binding to H3K4me2/3 (3) were known; but neither the underlying mechanisms nor the possible impact of SPOC1-expression levels on DDR or DNA repair have been described so far. We now propose an integrative mechanistic model of SPOC1’s function in altering chromatin structure, DDR and DNA repair (Figure 8). Accordingly, by SPOC1 binding to chromatin exposing H3K4me2/3, and/or different interaction partners, chromatin can be compacted by SPOC1’s ability to increase global or local levels of KAP-1, HP1-α, H3K9 KMTs and H3K9me3. These heterochromatin building factors can act in concert or independently in both gene repression and chromatin condensation (17,53,54).

Loss of SPOC1 results in the release of several factors and marks associated with chromatin condensation (H3K9 KMTs, H3K9me3, KAP-1), and thus promotes chromatin decondensation. SPOC1 dose-dependent chromatin condensation and decondensaton, previously shown by micrococcal nuclease digestion experiments (2), is consistent with this interpretation, and with the now demonstrated associated differences in DDR, DNA repair kinetics and cellular radiosensitivity. Moreover, the ATM-dependent recruitment to and accumulation of SPOC1 protein at heterochromatic repair foci and endogenous 53BP1 bodies/OPT domains further argue for SPOC1’s involvement in chromatin structural changes required for timely repair and choice of repair pathways. DSBs induced by low dose γ-IR in chromatin condensed locally and/or globally with the help of SPOC1 contribute to impaired activation of ATM and delayed expansion of γH2AX and 53BP1 at DSBs.

This interpretation is strongly supported by the observed reduced levels of pKAP-1(S824), an essential factor for decondensing and then repairing heterochromatin-associated DSBs (28). In contrast, upon SPOC1 depletion DSBs in decondensed chromatin are associated with increased levels of DDR transducers (pATM, γH2AX) and mediator 53BP1 involved in DNA repair. Chromatin decondensation as well as increased pATM are also consistent with the increased γH2AX and 53BP1 accumulation at DSBs and their repair, similar to that documented in human cells with deficits in other heterochromatin building factors (25). The SPOC1-dependent positive and negative modulation of DNA repair results in decreased and increased cellular radiosensitvity, which may be a consequence of SPOC1 also affecting the accuracy of DNA repair. The SPOC1 depletion-induced reduction in HR activity detected in the p53 positive, but not p53 negative HR reporter cells could be due in part, or entirely to the different p53 status. This assumption is strongly supported by the recent demonstration that DSB repair by HR is significantly compromised in the absence of DNA damage-induced phosphorylation of p53 by the concerted action of DNA-PK, ATM and ATR, as well as the well-documented effects of p53 on DNA repair mediated by its functions in regulating the cell cycle and gene transcription (55,56).

A strong argument for SPOC1 playing a role in DDR is the observed increase in chromatin-bound SPOC1 protein 30 min after γ-IR and a simultaneous decrease in the nucleoplasmic fraction. This demonstrates that nucleoplasmic SPOC1 responds to DNA damage early on by its increased recruitment to chromatin. However, unlike 53BP1 the SPOC1 protein did not detectably accumulate at DSB repair foci, nor did the nuclear distribution pattern visibly change 30 min after γ-IR. Therefore, we assume that the pan-nuclear increase of chromatin-bound SPOC1 represents an early response to DNA damage.

In contrast, ATM-dependent accumulation of SPOC1 was clearly observed at DSB-associated DNA repair foci at 8 h post-DSB induction, with gradually increasing SPOC1 and 53BP1 up to the end of the observation period (24 h). By this late time period post-γ-IR, the number of foci had decreased and their size had grown much larger, a feature characteristic of DSB repair occurring in heterochromatic regions (25). These findings are reminiscent of the distribution of pKAP-1(S824) post-γ-IR, which at early times is distributed in an pan-nuclear manner and only detectably accumulates late in foci formed at highly compacted chromatin (9).

Whether the DNA damage-induced changes in the distribution pattern of chromatin-bound SPOC1 are also mediated by ATM and/or Chk2 phosphorylation, as reported for pKAP-1(S824/473), is an open question. Alternatively, SPOC1 overexpression interfering with KAP-1 at S824 phosphorylation may be caused by KAP-1/SPOC1 protein–protein interaction-induced structural changes masking KAP-1 phosphorylation sites, combined with, or independently of SPOC1-induced alteration in ATM activity. SPOC1 may also affect KAP-1 dephosporylation, known to be mediated by phosphatase PP4 (57,58). In addition, the SPOC1 overexpression-induced higher level of chromatin-bound HP1-α may also affect KAP-1 phosphorylation, consistent with HP1’s known regulatory roles in both KAP-1 phosphoryation and dynamic chromatin structural changes occurring globally and locally at foci (59,60). Evidence for HP1 proteins playing a role in the response to γ-IR-induced IRIFs and DDR (27,31,32,61) supports our speculations. Whether SPOC1’s γ-IR-induced chromatin-binding pattern is associated with, or driven by changes in phosporylation or other post-translational modifications on SPOC1, and/or by corecruited factors involved in DDR is an interesting question that remains to be examined.

The inverse correlation between SPOC1-expression levels and the number of IRIF 30 min post-2 Gy γ-IR raised the question of whether this reflects altered sensitivity of DNA for DSB induction, subsequent DDR, or repair. The Comet assay performed with cells deficient in or overexpressing SPOC1 showed no difference, indicating that SPOC1 does not alter the radiosensitivity of DNA. This finding is consistent with increasing evidence for similar sensitivity of euchromatin and heterochromatin in forming DSBs (62,63). Therefore, we conclude that SPOC1 modulates the kinetics of DNA repair foci formation and DNA repair, but not the extent of initial damage. Since we failed to detect major changes in cell-cycle phase regulation upon SPOC1 depletion or overexpression (FACS analyses) and saw only minor changes in cell proliferation, we believe that the SPOC1-dependent differences in cellular radiosensitivity are triggered by SPOC1’s ability to differentially affect repair pathways, and thus also the accuracy of DNA repair.

The chromatin compaction upon SPOC1 overexpression (2) and the observed changes in IRIF kinetics are consistent with reports that DSBs in heterochromatin become fully accessible for sensors and mediators of DDR and repair only after partial chromatin relaxation and translocation to the periphery of heterochromatin (28,62,63). Proven or proposed key mediators of these processes include ATM-mediated KAP-1 phosphorylation (29), and epigenetic writers, readers and erasers (6,9,19,23,28,60,64). We have shown here that several of these mediators (KAP-1, HP1, ATM) are modulated by SPOC1, possibly explaining at the molecular level the observed SPOC1-mediated changes in the kinetics of IRIF formation, DSB repair and radiosensitivity.

KAP-1 is one of the best studied heterochromatin proteins and is phosphorylated in response to DNA damage (17,29). It accumulates as pKAP-1-S824 and mediates chromatin relaxation at IRIF in concert with chromatin remodeler CHD3 (23), which is essential for repairing DNA damage in heterochromatin. Our study revealed SPOC1-dependent modulation of chromatin-bound KAP1 in both γ-irradiated and non-irradiated cells. This change in KAP-1 levels may be due to SPOC1/KAP-1 protein interaction-triggered (de)stabilization, and presumably contributes to the SPOC1-dependent changes in chromatin (de)condensation (2).

Although sequence analysis revealed no evident KRAB-like domain, SPOC1 may act like KRAB containing proteins and other proteins shown to bind the RBCC domain of KAP-1 (17). If true, some of the many thousands of genes known to be targeted and transcriptionally altered *in vivo* by KAP-1 (65,66) could be cotargeted and regulated by SPOC1 in non-irradiated cells. However, the considerable cell-type-specific differences in both KAP1 and SPOC1 expression, the discrepancies between *in vitro/in vivo* data published for KAP-1-mediated recruitment and gene regulation, as well as open questions concerning the *in vivo* relevance of all KAP-1 protein interactions with KRAB/KRAB-like proteins, except for ZNF 274 (17), certainly make this a very demanding topic to investigate.

All HP1 isoforms, HP1-α, HP1-β and HP1-γ, are recruited to DSBs in human cells independently of H3K9 trimethylation (6,31). Therefore HP1 proteins are apparently important for DNA repair as well as their known function in higher order chromatin structure, and may act to stabilize loose ends and keep together sister chromatin in response to damage. However, HP1 proteins also have isoform-specific roles in DDR (6) and other biological processes (67). For instance, human cells overexpressing HP1-α and HP1-β, but not HP1-γ, show increased sensitivity to IR (68).

Our study corroborates this observation since SPOC1 overexpression increased levels of HP1-α and radiosensitivity. Whether the selective increase in HP1-α expression is due to protein stabilization enhanced by direct or indirect interaction with SPOC1 remains to be studied. Independently of the regulatory mechanism, increased HP1-α levels probably contribute to chromatin compaction and impaired DDR. HP1-α overexpression was recently reported in tumor tissues and proposed to play a crucial role in cancer prognosis (69). The inverse relationship of SPOC1-expression levels with cancer patient survival time (1) may be related to SPOC1-enhanced HP1-α expression.

The correlation between SPOC1 protein levels and H3K9 trimethylation as well as expression of several H3K9 KMTs implicates SPOC1 functions in both chromatin (de)condensation and DDR. H3K9me3 is not only an epigenetic mark characteristic of heterochromatin, but also the binding platform of HP1 and KAP-1, which collectively promote heterochromatin compaction and spreading (70,71).

Three lines of evidence indicate an important role for H3K9me3 in DDR and DNA repair. First, a sharp increase in H3K9me3 observed at I-SceI-induced DSBs is thought to transiently silence the gene to ensure that DNA repair is complete before transcription resumes, and/or compact the chromatin to mute DNA damage signaling stimulated by the initial chromatin opening (72). Second, binding of the acetyltransferase TIP60 via its CD to H3K9me3 at DSBs is required for its activation (24), and is a prerequisite for efficient ATM activation by acetylation. Third, the level of heterochromatin-associated histone modification H3K9me3 can account for >40% of mutation rate variation in human cancer cells (5).

The increase in HP1-α in SPOC1 overexpressing cells may interfere with TIP60 binding to H3K9me3 and its activation, possibly contributing to the observed reduction in activated ATM and delayed DDR. However, despite reduced H3K9me3 levels in SPOC1-depleted cells, ATM is apparently hyperactivated, perhaps indicating that its activity is independent of H3K9me3. Possible reasons for our observation are that ATM can also be activated in cells with low H3K9me3 levels (24). Furthermore, SPOC1 depletion, which promotes chromatin relaxation, may increase accessibility of H3K9me3 for TIP60, leading to increased ATM activation.

The histone H3K9 KMTs shown here to be modulated by SPOC1 (SETDB1, G9A, GLP) exist in a multisubunit complex that is destabilized by depleting the individual components (33). While indirectly confirming these findings, our data also demonstrate the importance of SPOC1’s interaction with this complex for its stability and activity.

Evidence provided here on SPOC1’s impact on DDR, DNA repair, and radiosensitivity may also be relevant for the recently reported function of SPOC1 in testicular germ stem-cell differentiation. In particular, both SPOC1 (4) and KAP-1 knockout (73) as well as a HP1-binding deficient KAP-1 mutant (74) all lead to progressive depletion of spermatogonial stem cells and their differentiated derivatives. The similar phenotypes of KAP-1 and SPOC1 knockout in testis argues for a cooperative physiological relationship *in vivo* in stem-cell maintenance and differentiation.

The presumed function of SPOC1 in oncogenesis (1) is consistent with its DDR modulating function shown here. Moreover, since SPOC1 is able to alter cellular radiosensitivity, it may be a relevant target for novel anti-cancer drugs that focus on manipulating differences in lysine methylation (75).

## AUTHOR CONTRIBUTION

A.M and H.W conceived and designed the experiments. A.M., H.S., K.R., L.F., V.B., S.A., M.K. and T.S. performed the experiments. A.M., E.S., S.A., S.K. and H.W. analyzed the data. A.H., A.M., E.S., M.K. and S.A. contributed reagents/materials/analysis tools. A.M. and H.W. wrote the paper.

## SUPPLEMENTARY DATA

Supplementary Data are available at NAR Online: Supplementary Figures 1–6 and Supplementary References [33,39,40].

## FUNDING

The Deutsche Krebshilfe and Müggenburg Foundation; HPI is supported by the Federal Ministry of Health and the Freie und Hansestadt Hamburg, Germany; Ait-Si-Ali group is supported by the Agence Nationale de la Recherche (ANR, French gvt); Association Française contre les Myopathies (AFM); Fondation Bettencourt-Schueller. Funding for open access charge: Heinrich-Pette-Institute, Leibniz-Institute for Experimental Virology.

*Conflict of interest statement*. None declared.

## Supplementary Material

Supplementary Data
