# SPOC1 modulates DNA repair by regulating key determinants of chromatin compaction and DNA damage response

**DOI:** 10.1093/nar/gkaa754

**Published:** 2020-09-05

**Authors:** Andreas Mund, Tobias Schubert, Hannah Staege, Sarah Kinkley, Kerstin Reumann, Malte Kriegs, Lauriane Fritsch, Valentine Battisti, Slimane Ait-Si-Ali, Anne-Sophie Hoffbeck, Evi Soutoglou, Hans Will

**Affiliations:** Heinrich-Pette-Institute, Leibniz-Institute for Experimental Virology, Department of General Virology, Martinistrasse 52, 20251 Hamburg, Germany; Heinrich-Pette-Institute, Leibniz-Institute for Experimental Virology, Department of General Virology, Martinistrasse 52, 20251 Hamburg, Germany; Heinrich-Pette-Institute, Leibniz-Institute for Experimental Virology, Department of General Virology, Martinistrasse 52, 20251 Hamburg, Germany; Heinrich-Pette-Institute, Leibniz-Institute for Experimental Virology, Department of General Virology, Martinistrasse 52, 20251 Hamburg, Germany; Heinrich-Pette-Institute, Leibniz-Institute for Experimental Virology, Department of General Virology, Martinistrasse 52, 20251 Hamburg, Germany; University Medical Center Hamburg, Clinic and Policlinic of Radiation Biology and Experimental Radiooncology, Laboratory of Radiobiology & Experimental Radiooncology, Martinistrasse 52, 20246 Hamburg, Germany; University Paris Diderot, Sorbonne Paris Cité, Laboratoire Epigénétique et Destin Cellulaire, UMR7216, Centre National de la Recherche Scientifique CNRS, 35 rue Hélène Brion, F-75013 Paris; University Paris Diderot, Sorbonne Paris Cité, Laboratoire Epigénétique et Destin Cellulaire, UMR7216, Centre National de la Recherche Scientifique CNRS, 35 rue Hélène Brion, F-75013 Paris; University Paris Diderot, Sorbonne Paris Cité, Laboratoire Epigénétique et Destin Cellulaire, UMR7216, Centre National de la Recherche Scientifique CNRS, 35 rue Hélène Brion, F-75013 Paris; Department of Cancer Biology, Institut de Génétique et de Biologie Moléculaire et Cellulaire (IGBMC), CNRS UMR 7104, INSERM U 596, Centre Universitaire de Strasbourg, 67404 Illkirch CEDEX, France; Department of Cancer Biology, Institut de Génétique et de Biologie Moléculaire et Cellulaire (IGBMC), CNRS UMR 7104, INSERM U 596, Centre Universitaire de Strasbourg, 67404 Illkirch CEDEX, France; Heinrich-Pette-Institute, Leibniz-Institute for Experimental Virology, Department of General Virology, Martinistrasse 52, 20251 Hamburg, Germany

*Nucleic Acids Research*,2012, 40(22): 11363–11379, https://doi.org/10.1093/nar/gks868

The authors would like to apologize for a mistake made during the construction of Figure 6. The HP1-β, HP1-γ, and H3 negative western blot results of the soluble-rich fraction were accidental masked with the same noise background. This error happened during figure arrangement. A new Figure 6 where the noise background has been removed, revealing the original negative data behind it, is provided below. This error does not change the conclusion of the western blot analysis nor the scientific conclusion of the article.

**Figure 6.**
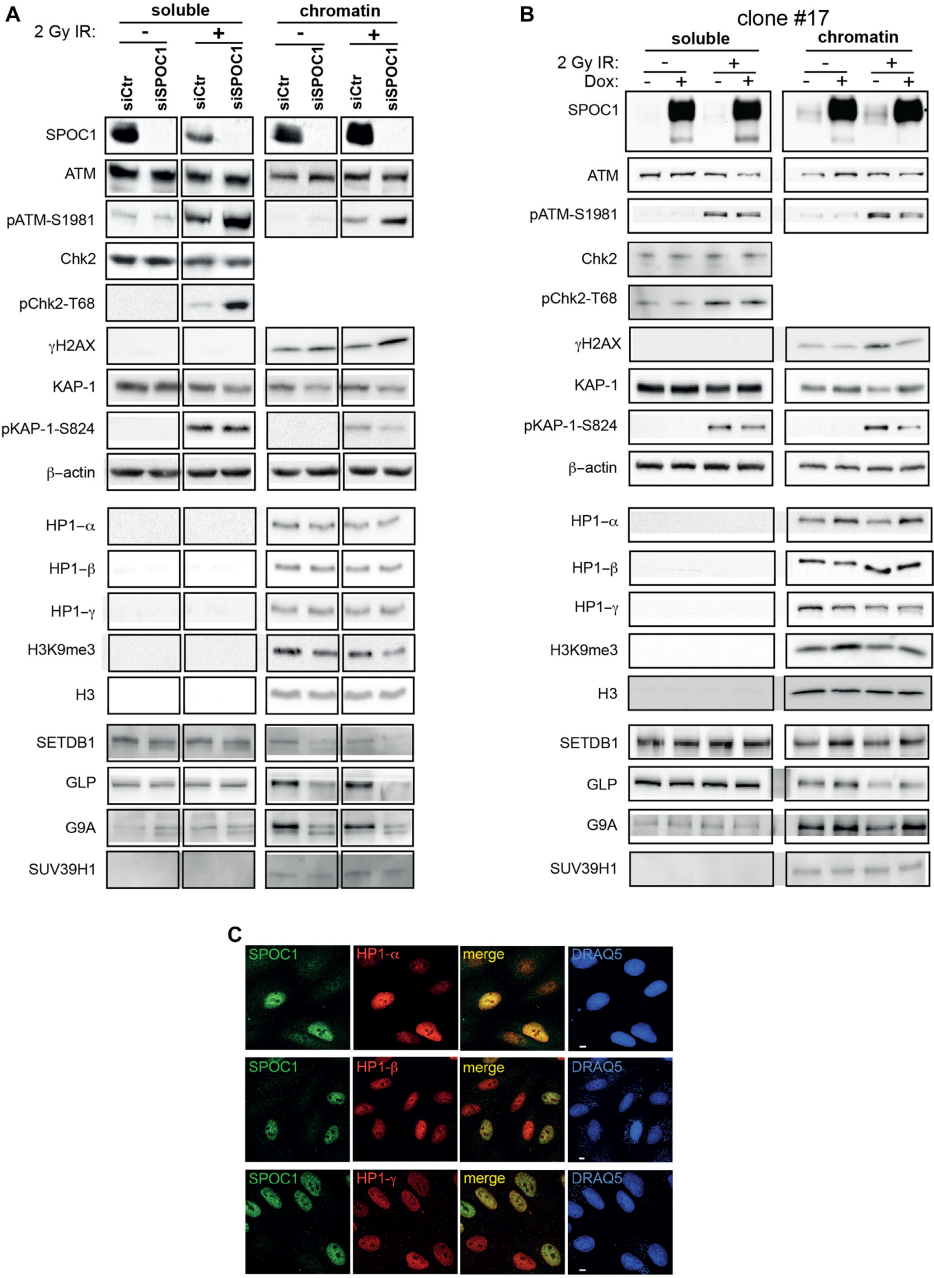
SPOC1 levels modify expression, modification and localization of proteins involved in chromatin (de)compaction and DDR. Immunoblots of soluble and chromatin-rich fractions from untreated (-) and irradiated cells collected 30 min post-2 Gy g-IR (+) analyzed with Abs against the indicated proteins. (**A**) SPOC1-siRNA transfected cells show less SPOC1 protein than control CV1 cells. (**B**) Dox-treatment strongly enhances SPOC1 levels in CV1 cell line clone #17. (**A** and **B**) Immunoblot signal intensities for different cellular proteins detected in the corresponding cellular lysates show specific changes in expression of some but not all, depending on the soluble or chromatin fraction, SPOC1-expression levels and/or g-IR. (**C**) Confocal immunofluorescence analysis of mixed CV1 cells over and underexpressing SPOC1 immunostained for HP1-a (top), HP1-b (middle) and HP1-g (lower panels) only showed enhanced HP1-a expression in SPOC1-overexpressing cells. SPOC1 was immunostained with antibody CR56, the HP1 proteins with the antibodies given in ‘Materials and Methods’ section, and DNA was stained by DRAQ5. Scale bars = 5 mm.

## Notes

Present address: Hans Will, Department of General Virology, Heinrich-Pette-Institute, Leibniz-Institute for Experimental Virology, Martinistr. 52, 20251 Hamburg, Germany.

