# Supplementary material for: SPOC1 modulates DNA repair by regulating key determinants of chromatin compaction and DNA damage response
Source: Nucleic Acids Res. 2012 Oct 2;40(22):11363–79. doi: 10.1093/nar/gks868 (PMC3526275; doi:10.1093/nar/gks868)
Supplement: Supplementary Data [file supp_40_22_11363__index.html]

SPOC1 modulates DNA repair by regulating key determinants of chromatin compaction and DNA damage response — SPOC1 modulates DNA repair by regulating key determinants of chromatin compaction and DNA damage response — Supplementary Data 

# SPOC1 modulates DNA repair by regulating key determinants of chromatin compaction and DNA damage response

## Supplementary Data

files

**Files in this Data Supplement:**

- Supplementary Data - pdf file
