# Supplementary material for: SPOC1 modulates DNA repair by regulating key determinants of chromatin compaction and DNA damage response
Source: Nucleic Acids Res. 2012 Oct 2;40(22):11363–79. doi: 10.1093/nar/gks868 (PMC3526275; doi:10.1093/nar/gks868)

Mund et al. Supplementary Data

Supplementary Figure Legends:

Supplementary Figure 1. SPOC1 levels do not change the amount of initial DNA damage after 2 Gy γ-IR

(A) Cells with siRNA-mediated knockdown of SPOC1 expression and control siRNA treated cells, as well as (B) two different cell lines with Dox-inducible SPOC1 overexpression (#17, #23) were irradiated with 2 Gy on ice and harvested immediately. The olive tail moment was measured using the Comet analysis software VisComet 4.0 (Impuls Bildanalyse GmbH, Gilching). Sixty comets were scored for each treatment in two independent experiments. Proportions of cells in G1/S/G2 on the corresponding FACS profiles shown on the right were estimated using FACSDiva v6 software.

Supplementary Figure 2. Constructs chromosomally integrated in H1299.EJ or H1299.GC cell lines used as reporters for NHEJ or HR

Both cell lines contain chromosomally integrated EGFP that is only expressed after I-SceI-mediated cleavage and repair. (A) In H1299.EJ cells the EGFP construct contains an out of frame insert flanked by two SceI sites. Cleavage with I-SceI removes the insert, leading to EGFP expression upon repair of the DSB by NHEJ. (B) The H1299.GC cells contain a stably integrated mutant EGFP harbouring two SceI cleavage sites in its coding sequence preventing its expression. Upon I-SceI cleavage the mutant EGFP can be repaired by HR and converted into wild type EGFP. (A and B) schemes are adapted from (39, 40).

Supplementary Figure 3. SPOC1 expression level does not affect EGFP expressed from a plasmid in NHEJ and HR reporter cells

(A to C) Expression of EGFP in NHEJ (H1299.EJ; A and B) and HR (H1299.EJ; C) reporter cells as monitored by flow cytometry in response to altered SPOC1 expression. Immunoblot analysis of SPOC1 expression levels in all samples using SPOC1 mAbs (A, C=6F6; B=1D3) or GAPDH Ab demonstrates successful SPOC1 knockdown (A, C) and similar SPOC1wt and mutant protein expression levels (B). (A and C) Cells were co-transfected with pEGFP and siRNA against SPOC1 or control siRNA as indicated. Forty eight hours later EGFP expression was measured in (A) H1299.EJ and (C) H1299.GC cells using flow cytometry. No significant influence of SPOC1 knockdown on EGFP gene expression from the co-transfected plasmid was apparent. (B) H1299.EJ cells were co-transfected with pEGFP and expression plasmids, encoding either SPOC1wt or SPOC1 PHD (PHDmt) or NTD (deltaN) mutants. Forty eight hours later EGFP expression was measured using flow cytometry. Overexpression of SPOC1wt or mutant proteins showed no significant effect on EGFP gene expression from the cotransfected plasmid.

Supplementary Figure 4. Growth kinetics of CV1 cells with and without altered SPOC1 expression

(A-C) Growth kinetics of wt CV1 cells compared to three independent CV1 cell lines (clones #14, #17 and #23) with low (-Dox) or high (+Dox) level FLAG-SPOC1 protein expression. Reduced cell proliferation was observed from day two onwards, post plating in cells, either as low leaky or much stronger at high SPOC1 Dox-induced overexpression in CV1 cell lines compared to control CV1 cells. (D) Comparative

growth kinetics of CV1 cells transfected with control siRNA or SPOC1 specific siRNA reveals only a very minor difference in cell proliferation. Cell numbers were determined in triplicate from three independent dishes and given as mean values +/- SD. For SPOC1 and FLAG-SPOC1 expression levels in the different cells see Figure 3A, B.

Supplementary Figure 5. SPOC1 levels modify expression and posttranslational modification of proteins involved in chromatin (de)compaction and DDR.
(A) Immunoblots of soluble and chromatin-rich fractions from non-irradiated (-) as well as from CV1 cell line #23, collected 30 min post 2 Gy γ-IR (+), analyzed with the indicated Abs. Dox-treatment strongly enhances SPOC1 levels in CV1 cell lines. Comparison of immunoblot signal intensities for different cellular proteins analysed with the indicated antibodies and the cellular lysates shows specific changes in expression of some, but not all of them in the soluble or chromatin fraction, and depending on or independent of SPOC1 expression levels and/or γ-IR. (B) Confocal microscopy analysis of a mixture of CV1 cells with endogenous or enhanced SPOC1 expression, as evident by SPOC1 immunostaining. Double immunostaining of these cells for HP1-α (top), HP1-β (middle), and HP1-γ (lower panels) shows only enhanced HP1-α expression in SPOC1-overexpressing cells. DNA was immunostained by DRAQ5. Scale bars=5 µm.

Supplementary Figure 6. SPOC1 interacts with H3K9 KMTs in the absence of DNA and RNA.
(A) Immunoblot analysis of the FLAG-HA-tagged-G9A containing H3K9 KMT protein complex affinity purified as described (33), except that before the FLAG-HA double immunopurification steps, nuclear extracts were treated with a mixture of Turbo DNase I (Turb.) and RNase A (lane 4), or only Turbo DNase I (lane 3). The profil of DNA extracted from the treated nuclear extracts is shown in the right panel. B. SPOC1 was immunoprecipitated as in Figure 7 (anti-HA Abs were used as a negative control), except that before the immunopurification steps, nuclear extracts were treated with a either mixture of MNase and EtBr (B) or DNase and 150 µg of RNase (C). The profil of DNA extracted from the treated nuclear extracts is shown in the right panel.

Supplementary References

33. Fritsch, L., Robin, P., Mathieu, J.R., Souidi, M., Hinaux, H., Rougeulle, C., Harel-Bellan, A., Ameyar-Zazoua, M. and Ait-Si-Ali, S. (2010) A subset of the histone H3 lysine 9 methyltransferases Suv39h1, G9a, GLP, and SETDB1 participate in a multimeric complex. Mol Cell, 37, 46-56.

39. Kriegs, M., Kasten-Pisula, U., Rieckmann, T., Holst, K., Saker, J., Dahm-Daphi, J. and Dikomey, E. (2010) The epidermal growth factor receptor modulates DNA double-strand break repair by regulating non-homologous end-joining. DNA Repair (Amst), 9, 889-897.

40. Mansour, W.Y., Schumacher, S., Rosskopf, R., Rhein, T., Schmidt-Petersen, F., Gatzemeier, F., Haag, F., Borgmann, K., Willers, H. and Dahm-Daphi, J. (2008) Hierarchy of nonhomologous end-joining, single-strand annealing and gene conversion at site-directed DNA double-strand breaks. Nucleic Acids Res, 36, 4088-4098.

Mund et al Supplementary Figures:

Supplementary Figure 1

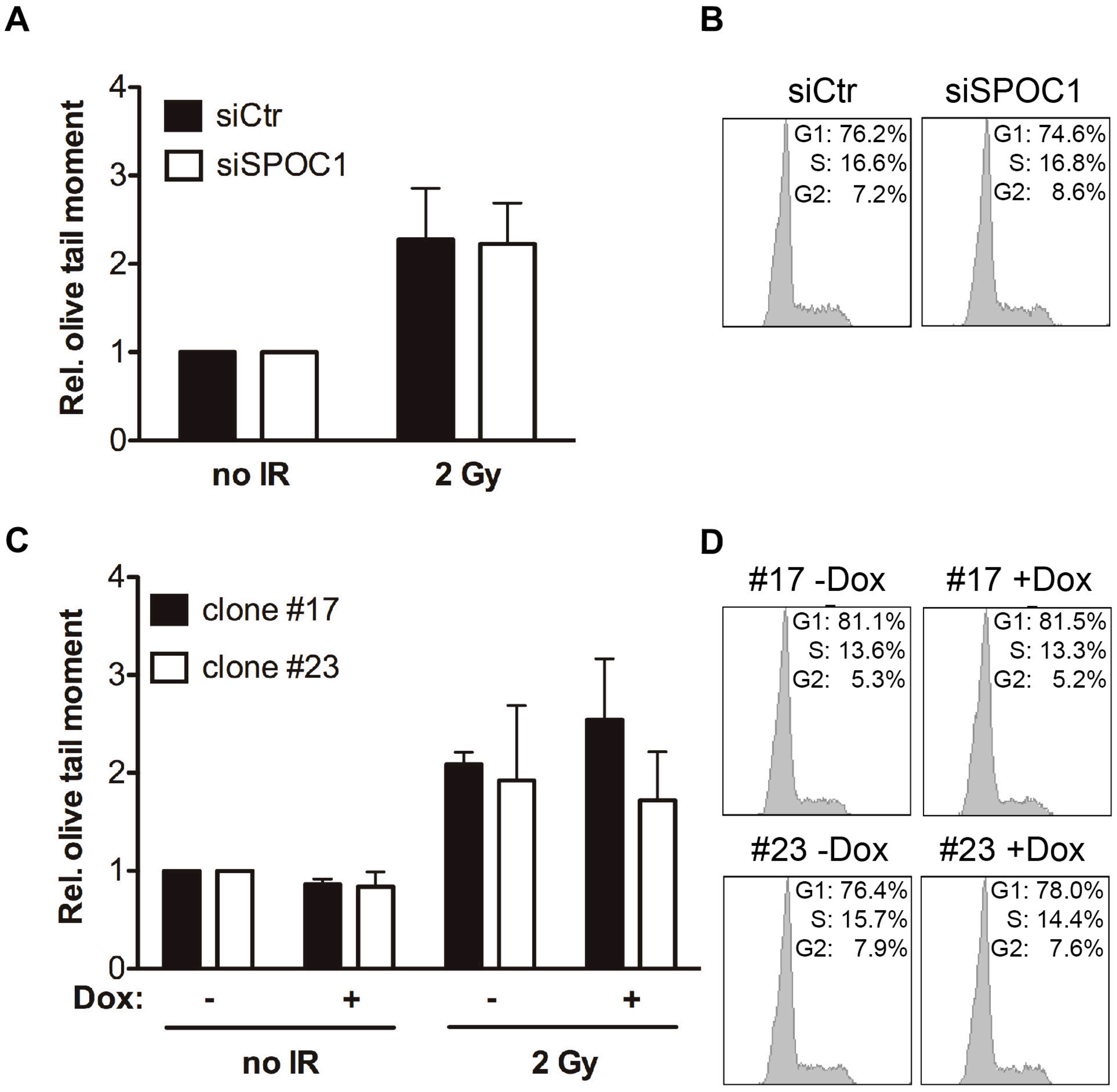

Supplementary Figure 2

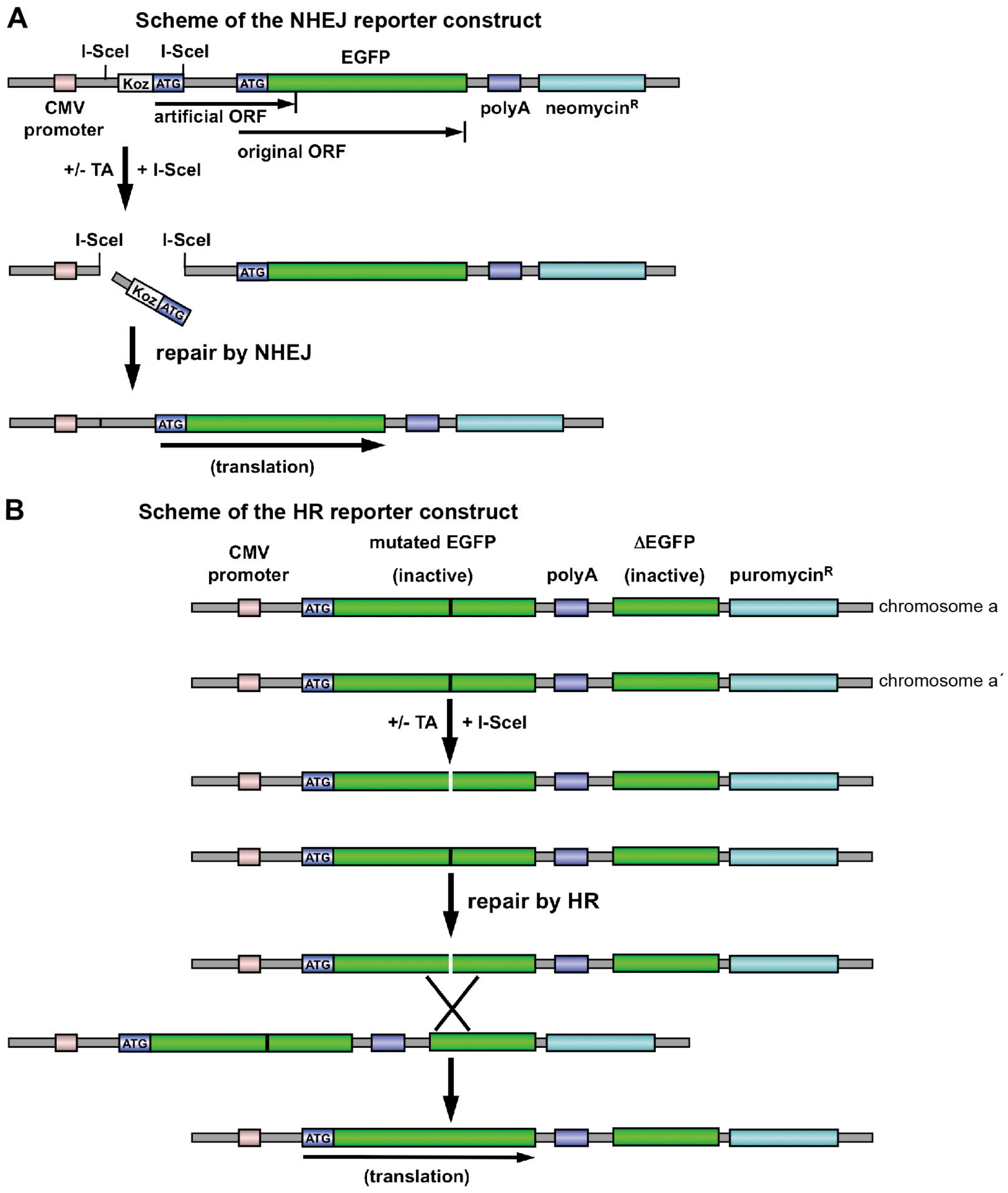

Supplementary Figure 3

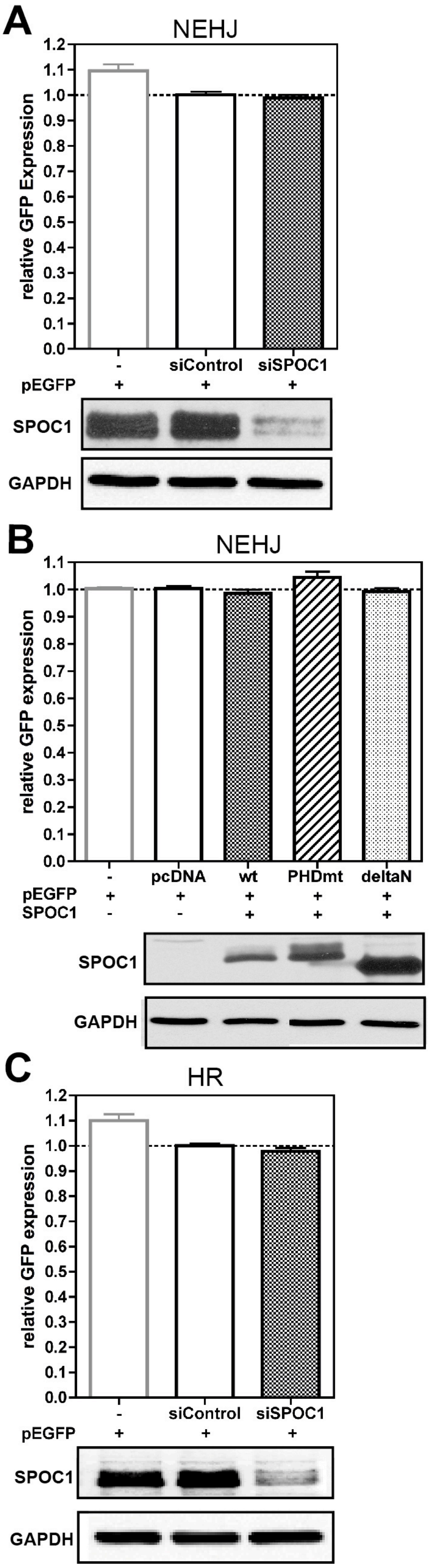

Supplementary Figure 4

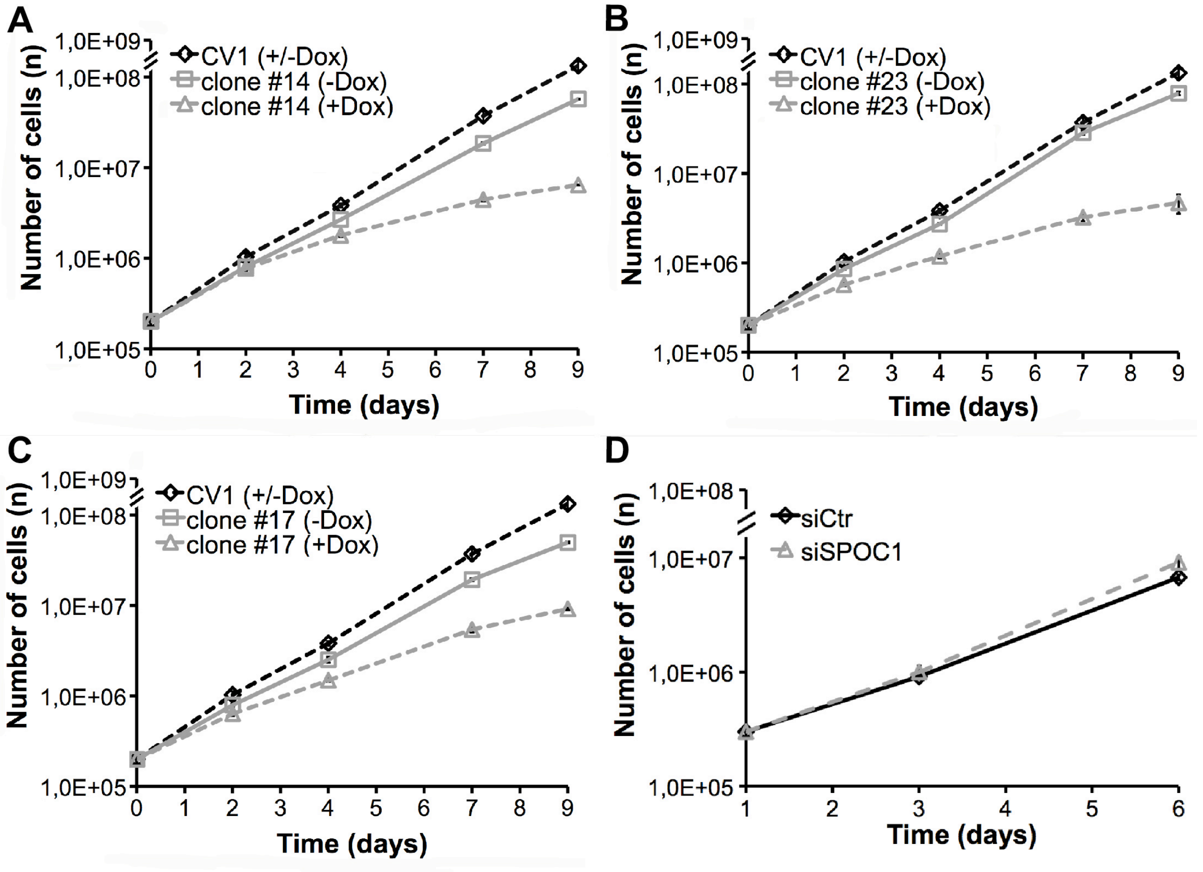

Supplementary Figure 5

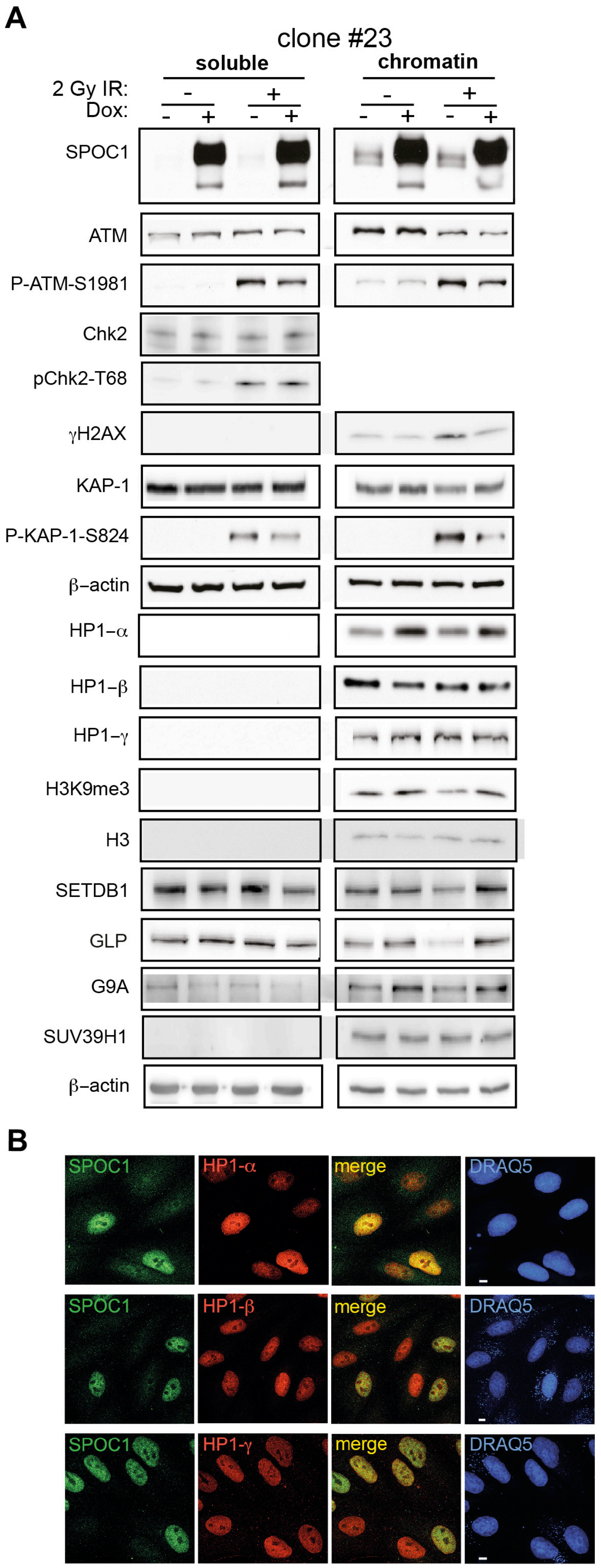

Supplementary Figure 6

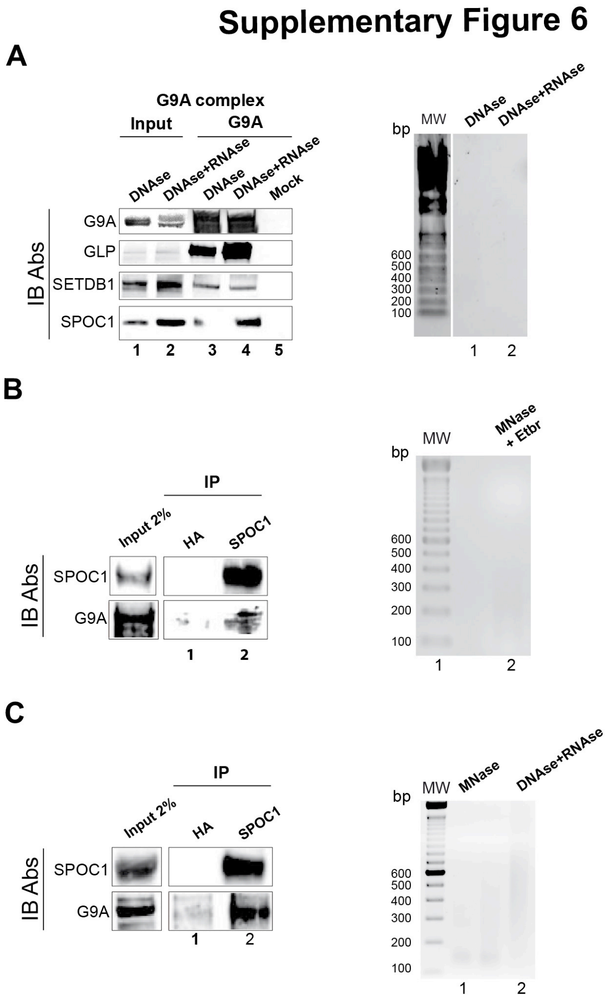

Supplement: Supplementary Data [file supp_gks868_nar-00787-m-2012-File010.pdf]
